# Tuning the Growth of Chiral Gold Nanoparticles Through Rational Design of a Chiral Molecular Inducer

**DOI:** 10.1021/acs.nanolett.3c02800

**Published:** 2023-10-25

**Authors:** Kyle Van Gordon, Sandra Baúlde, Mikhail Mychinko, Wouter Heyvaert, Manuel Obelleiro-Liz, Alejandro Criado, Sara Bals, Luis M. Liz-Marzán, Jesús Mosquera

**Affiliations:** † CIC biomaGUNE, Basque Research and Technology Alliance (BRTA), 20014 Donostia-San Sebastián, Spain; ‡ Universidade da Coruña, CICA−Centro Interdisciplinar de Química e Bioloxía, Rúa as Carballeiras, 15071 A Coruña, Spain; § EMAT and NANOlab Center of Excellence, University of Antwerp, B-2020 Antwerp, Belgium; ∥ EM3Works, Spin-off of the University of Vigo and the University of Extremadura, PTL Valladares, 36315 Vigo, Spain; ⊥ Biomedical Networking Research Center, Bioengineering, Biomaterials and Nanomedicine (CIBER-BBN), 20014 Donostia-San Sebastián, Spain; # Ikerbasque, 48009 Bilbao, Spain; ∇ Cinbio, Universidade de Vigo, 36310 Vigo, Spain

**Keywords:** chiral nanoparticles, gold nanoparticles, circular dichroism, plasmonic chirality, electron tomography

## Abstract

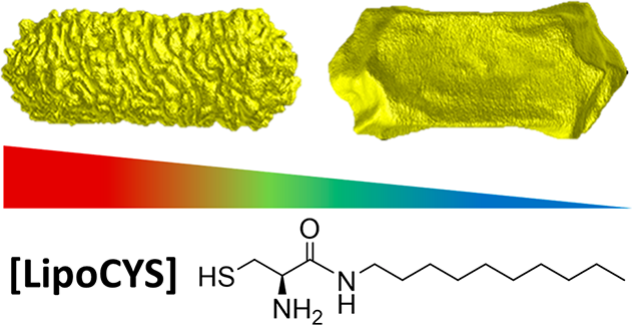

The bottom-up production of chiral gold nanomaterials holds great potential for the advancement of biosensing and nano-optics, among other applications. Reproducible preparations of colloidal nanomaterials with chiral morphology have been reported, using cosurfactants or chiral inducers such as thiolated amino acids. However, the underlying growth mechanisms for these nanomaterials remain insufficiently understood. We introduce herein a purposely devised chiral inducer, a cysteine modified with a hydrophobic chain, as a versatile chiral inducer. The amphiphilic and chiral features of this molecule provide control over the chiral morphology and the chiroptical signature of the obtained nanoparticles by simply varying the concentration of chiral inducer. These results are supported by circular dichroism and electromagnetic modeling as well as electron tomography to analyze structural evolution at the facet scale. Our observations suggest complex roles for the factors involved in chiral synthesis: the chemical nature of the chiral inducers and the influence of cosurfactants.

One does not need to look beyond their own hands to realize that chirality, a property that describes objects lacking internal or mirror symmetry, is ubiquitous. Even a polarized plane of light can itself be considered chiral, possessing right- and left-handed components.^1^ The field of chiral plasmonics takes advantage of an intersection of phenomena related to the interactions of matter with light. Near-field electromagnetic enhancements deriving from the resonance of oscillating conduction electrons (surface plasmons) in metal nanomaterials make the sensing of chiral small molecules and biomolecules using circularly polarized light demonstrably viable.^2−5^ Furthermore, the observations that the surface plasmons of chiral structures are also chiral, and that enhancement effects are collectively applied to the structure as a whole,^6^ have encouraged advances in the development of chiral plasmonic nanomaterials.

Lending to the adaptable nature of plasmonic nanoparticle (NP) synthesis methods^7,8^ and the wide range of chiral templates,^9,10^ several types of plasmonic NPs have been reported and a wider variety is expected.^11^ Chiral nanomaterials have already found relevant applications, mainly due to their strong circular dichroism (CD), and their specific interactions with chiral biomolecules, including molecular sensors^12,13^ and immunotherapy.^14,15^ Further expansion of such applications largely depends on our ability to tailor the chiral intricacy of the nanomaterial and to understand its influence on the resulting optical activity and spectral range. Consequently, the development of synthetic methodologies capable of yielding chiral nanomaterials with novel morphologies holds paramount importance for emerging areas such as nanomedicine, enantioselective catalysis, and even nanorobotics. In these applications, control over particle size is highly relevant, regarding the prominence of absorption or scattering in the optical response.

Arrangement of a nonchiral material into a configuration that imparts chiral properties in a bottom-up approach is foundational for the flexible and reproducible design of chiral nanomaterials.^16−18^ More challenging has been the colloidal synthesis of plasmonic nanomaterials with a chiral morphology at the single-particle level. Seeded-growth synthesis has emerged as an efficient methodology for the preparation of chiral nanostructures with a high optical activity. Among various other parameters, the geometry of the NP seed largely determines the final morphology of the resulting chiral NPs; here we focus on the use of gold nanorods (Au NRs) as seeds.^19^ Recent reports have shown that chiral seeded growth on Au NPs can be achieved through several alternative pathways, according to the chemical nature of the molecular inducer of dissymmetry.

One synthetic pathway results in the formation of plasmonic nanorods with twisted geometry (Figure 1a).^20^ This method was initially applied to other NP seed morphologies,^21,22^ using amino acids (such as cysteine) or short peptides containing the same amino acid, as the dissymmetry inducers. Asymmetric growth on the plasmonic seeds has been claimed to be induced by the enantioselective adsorption of chiral inducers on high-index facets with atomic-scale chirality. This preferential interaction with a particular chiral facet promotes the kinetic growth of crystallographic facets with the opposite handedness, ultimately resulting in the formation of twisted shapes.^21−23^ The same strategy has been applied to prepare several types of twisted Au NRs by the overgrowth of preformed achiral Au NRs. However, the outcome of the overgrowth process is significantly influenced by the experimental conditions employed. Our research team reported a cysteine-mediated multistep growth method, enabling the synthesis of 4-fold-twisted gold nanorods with a *g*-factor reaching values as high as 0.10.^20^ In contrast, Wu et al. achieved the fabrication of helical plasmonic nanorods through the synergistic combination of cysteine and the achiral molecule 4-aminothiophenol, in the presence of a significant amount of Ag^+^ ions, yielding a *g*-factor of 0.04.^24^ However, the specific role of the various additives has yet to be determined.

**Figure 1:**
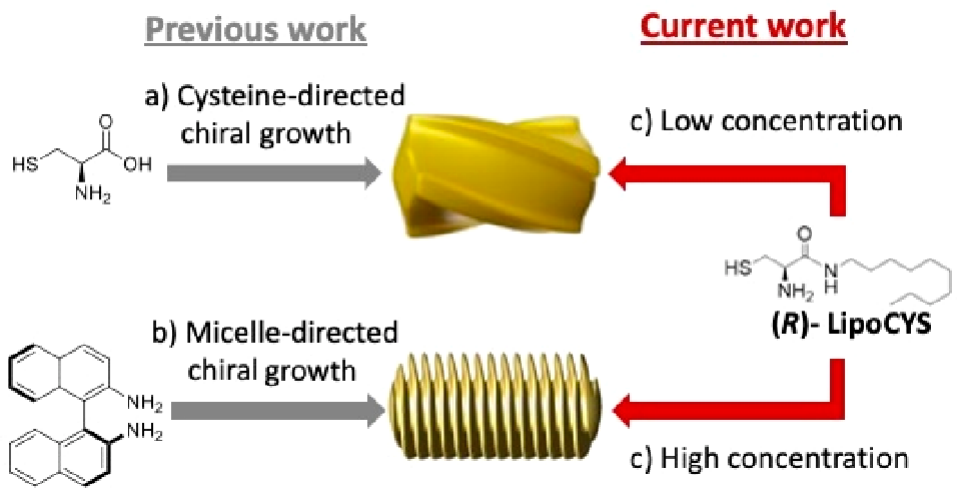
Schematic view of the mechanisms for seeded growth of chiral nanorods. (a) Cysteine as a chirality inducer, resulting in twisted NRs. (b) Chiral growth directed by micelles containing BINAMINE as cosurfactant, resulting in a wrinkled particle structure. The use of LipoCYS (c) is proposed to bridge both growth mechanisms.

A second generic synthetic protocol is known as micelle-directed chiral growth,^25,26^ in which the presence of a chiral cosurfactant leads to helical micelles wrapped around the Au NR seeds. Such helical micelles act as templates during seeded growth, resulting in a dense array of quasihelical wrinkles around a central NR (Figure 1b). Despite the wide variety of potential chiral cosurfactants, only one example has been reported so far, using 1,1′-bi(2-naphthylamine) (BINAMINE) as the chiral inducer, alongside a primary surfactant (cetyltrimethylammonium chloride, CTAC), to induce chiral seeded growth (Figure 1b). BINAMINE is a molecule with axial chirality, containing an aromatic hydrophobic region that can be inserted in the CTAC micelles and two amino groups that assist binding onto the Au surface. On this basis, it has been postulated that the BINAMINE plays a dual role, inducing both the formation of chiral wormlike micelles and a stronger interaction between the elongated micelle and the seed surface through amine groups. This interaction between the elongated micelles and the seed is required for the formation of a micellar template that can promote the growth of steep wrinkles and the stabilization of such morphological features. Although chiral growth on Au NR seeds with various dimensions has been demonstrated,^26^ high-aspect-ratio NRs are typically used to enhance the formation of helical features and maximize the chiroptical activity.

Emboldened by the success of chiral synthesis using amino acids and inspired by the putative micellar template growth mechanism, we explored the role of a purposely devised chiral molecule consisting of a modified cysteine, (*R*)-2-amino-*N*-decyl-3-mercaptopropanamide, which we term (*R*)-LipoCYS (Figure 1; preparation and characterization details are provided in the Supporting Information), as the chiral inducer for the seeded growth of gold nanorods. The presence of a cysteine-like headgroup and a longer hydrophobic tail was foreseen to provide a dual function, allowing high affinity for both the gold surface and the micellar system. The choice of gold nanorods as achiral seeds provides a larger surface area per particle for adsorption of the cysteine moiety, while capitalizing on the potential for micelle formation, maximizing their elongation and promoting the growth of distinct chiral features. We show that LipoCYS is a versatile inducer that can produce both twisted and wrinkled chiral plasmonic nanoparticles as described above, depending on its concentration. At relatively low concentrations, LipoCYS behaves similarly to cysteine and generates twisted gold nanorods. In contrast, high concentrations of LipoCYS lead to the formation of NPs with well-defined helical wrinkles, typically obtained through micelle-directed growth. The observed morphological changes are also reflected in distinct optical features with varying intensities and spectral positions.

To evaluate the performance of LipoCYS as a chiral inducer, we implemented experimentally the growth of preformed gold nanorods (142 ± 10 nm long, 32 ± 2 nm thick; see Figure S1 and the Supporting Information for details) using CTAC as a surfactant and ascorbic acid (AA) as a reducing agent. This protocol has been previously applied to produce colloidal dispersions of chiral nanoparticles with an intense chiroptical response using BINAMINE as the chiral inducer.^25^ As a means to monitor the quality of the produced chiral NRs, circular dichroism (CD) spectroscopy was used to quantify the chiroptical response, i.e. the unequal extinction of left- and right-handed circularly polarized light.^27^ Additionally, to characterize surface wrinkles and other geometrical features, high-angle annular dark-field scanning transmission electron microscopy (HAADF-STEM) was combined with tomography to investigate the morphology and structural features in 3D, which can hardly be discerned in conventional 2D HAADF-STEM images. Through the modification of experimental parameters, we aimed at maximizing the *g-*factor, while monitoring the resulting structural evolution.

A gradual increase of the LipoCYS concentration (keeping all other variables constant) resulted in a spectral red shift of the chiroptical signature and a corresponding change in particle morphology (Figure 2). The NPs obtained at 20 μM of LipoCYS have an average length of 186 ± 8.0 nm and an average outer diameter of 78.6 ± 5.5 nm. Regarding the *g-*factors, the maximum value is obtained at around 620 nm, with values of 0.025 for (*R*)-LipoCYS and 0.009 for (*S*)-LipoCYS. Figure 2A illustrates that larger *g*-factors are consistently achieved with the *R* enantiomer, which may be related to inherent challenges associated with thiolated molecules and their potential oxidation by atmospheric oxygen.

**Figure 2:**
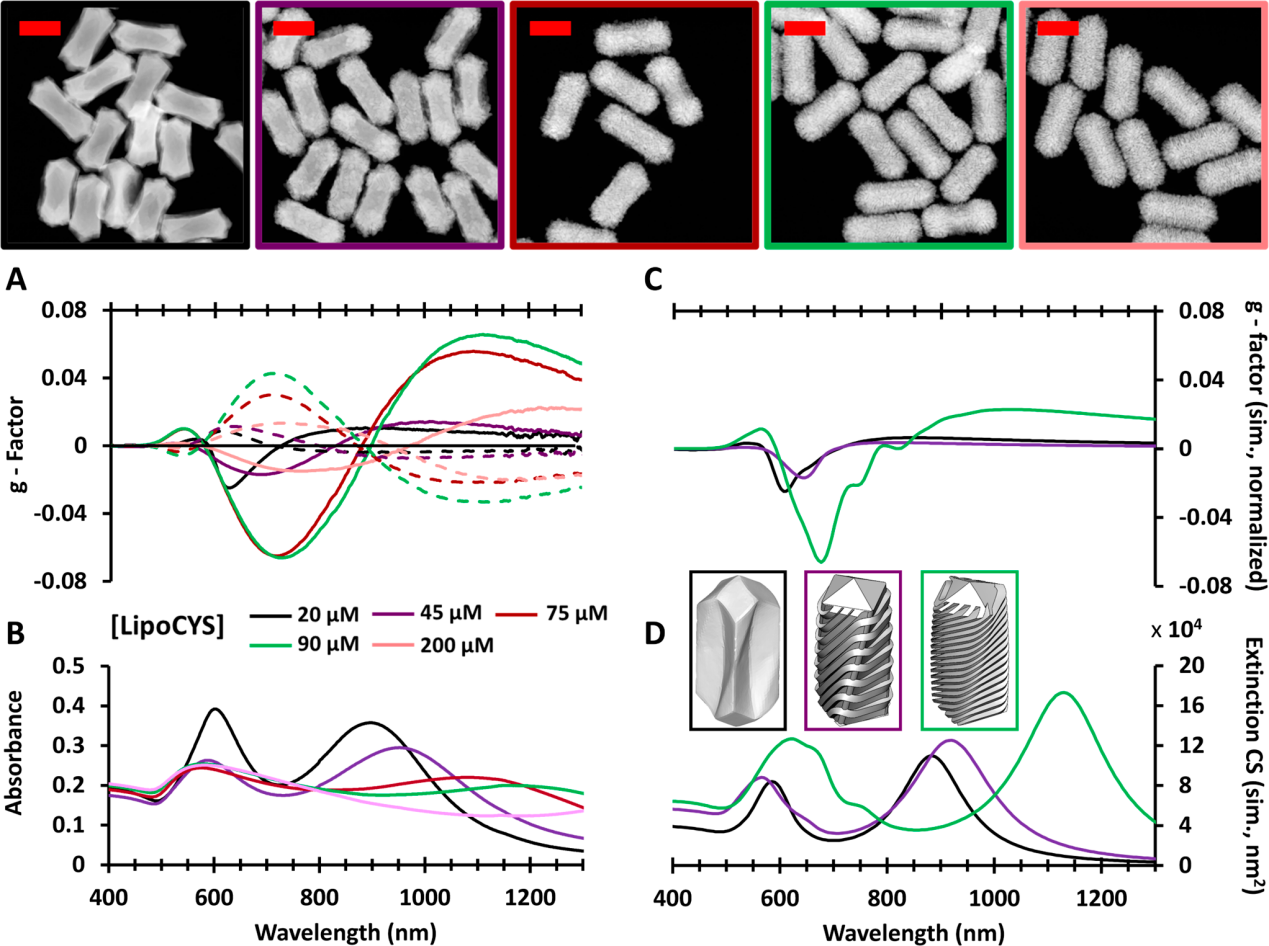
Circular dichroism (*g-*factor) spectra (A) and absorbance spectra (B) for chiral Au NR colloids prepared using different [LipoCYS], as labeled. Solid and dashed lines correspond to results from particles synthesized with the *R* and *S* enantiomers of LipoCYS, respectively. Shown above are HAADF-STEM images of chiral particles synthesized with (*R*)-LipoCYS; image outlines are color-coded to match the legend of the plots. Scale bars: 100 nm. Simulated circular dichroism (C) and extinction (D) spectra for three models resembling the morphology of selected experimental samples (as indicated by the color code). Simulations were based on SIE-MoM (see text and the Supporting Information for details).

A plateau in the maximum obtained *g-*factor was observed for wrinkled particles synthesized with 90 μM LipoCYS (equivalent to a ratio of 1:486 LipoCYS to CTAC molecules). These particles have average dimensions of 190 ± 9.9 nm × 75.9 ± 3.7 nm and maximum *g-*factors (at 720 nm) of 0.066 for (*R*)-LipoCYS and 0.043 for (*S*)-LipoCYS. Additional wrinkling on particles at higher LipoCYS concentration resulted in weaker chiroptical activity, which might be related to a lower degree of order in surface topography at higher concentrations or a complex directional geometry developing at the particle tips.^28^ This hypothesis is supported by the results of a quantitative helicity analysis (Figure S2) based on HAADF-STEM tomography, which confirms the direct correlation between the handedness of the studied NPs and the type of enantiomer used during the synthesis.^29^ For the NRs prepared with the highest LipoCYS concentration (200 μM), a less defined helicity plot was obtained, indicating undefined chirality (Figure S3).

As additional evidence behind the correlation between the obtained morphologies and the plasmonic optical activity, we carried out electromagnetic simulations based on the surface-integral equations (SIEs) discretized by the method of moments (MoM) (see Figure S4 and the Supporting Information for details).^30^ We used 3D models, based on the electron tomography reconstructions in Figures 3 and 4, that resembled chiral NRs obtained with three different LipoCYS concentrations. The simulated CD spectra (Figure 2C) were found to agree with the experimental trend in terms of both wavelength and relative intensity of the plasmonic CD bands. The increased and red-shifted *g-*factor bands observed for increasing LipoCYS concentrations are related to the formation of well-defined wrinkles, in agreement with a detailed computational analysis reported elsewhere.^31^ The effect of disorder in the wrinkled structure (for the highest LipoCYS concentrations) was also accounted for in the simulations, indeed revealing a loss of CD signal when the surface features are disordered (Figure S5).

**Figure 3:**
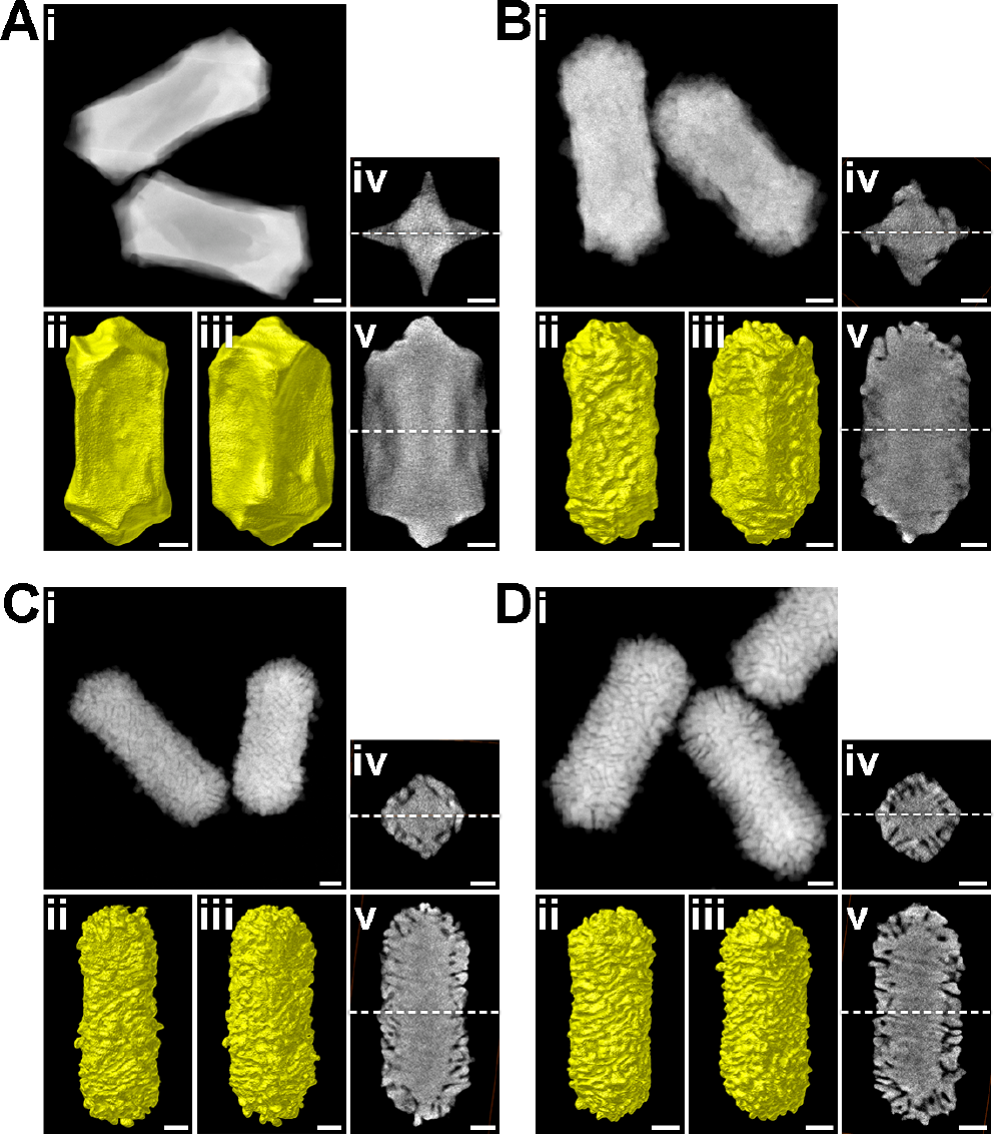
Morphological characterization of Au NPs obtained by increasing the concentration of (*R*)-LipoCYS (A, 20 μM; B, 45 μM; C, 75 μM; D, 90 μM) during chiral overgrowth. The morphological characterization for each sample includes (i) HAADF-STEM image of several representative nanoparticles, (ii, iii) Visualizations of the 3D reconstructions, presented along different viewing angles (oriented 45° relative to each other), and (iv, v) selected orthoslices extracted from the 3D reconstructions, perpendicular to the longitudinal and transverse axes, at the center of the NRs. White dashed lines represent the relative positions of slices shown in (iv) and (v). All scale bars are 25 nm.

**Figure 4:**
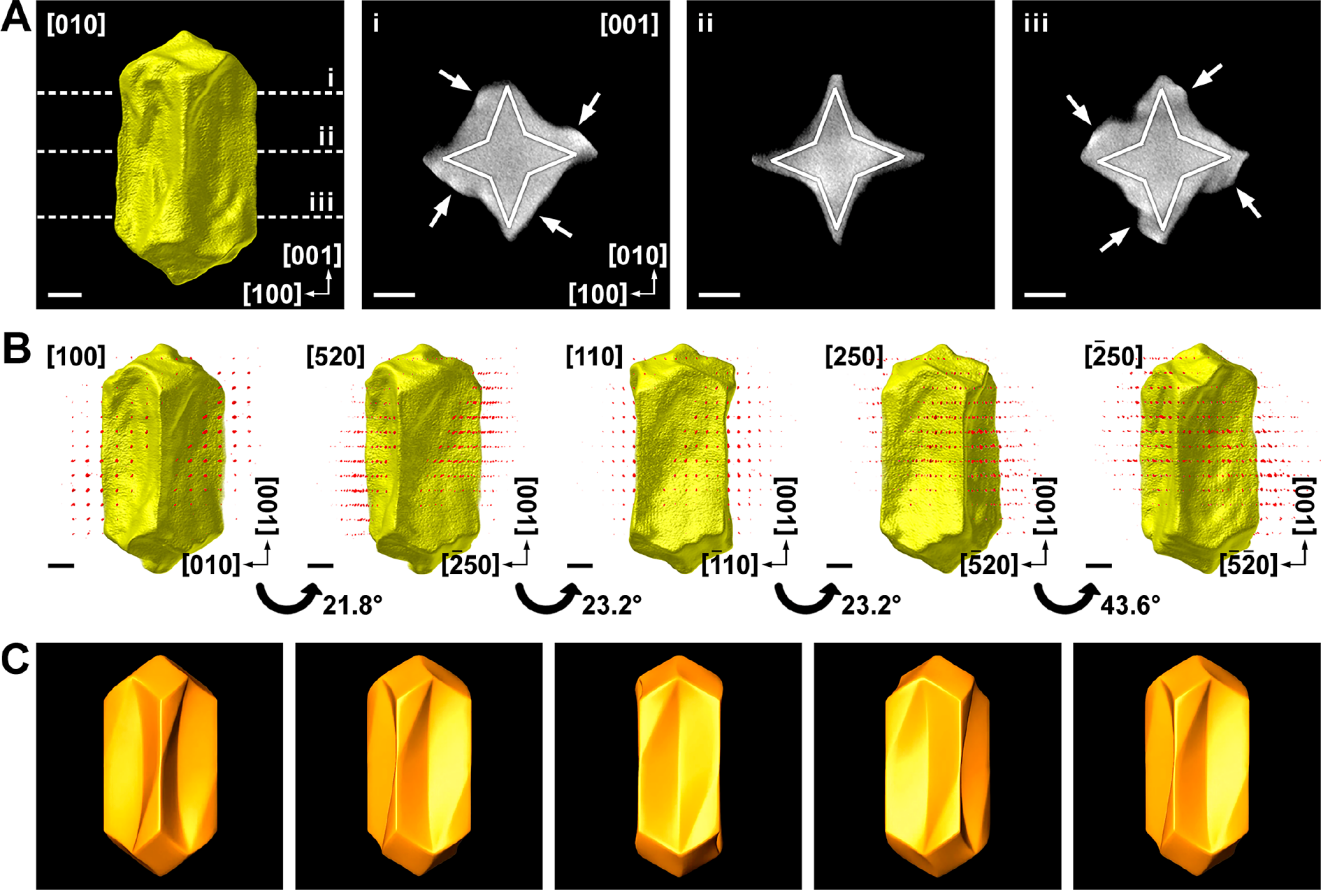
(A) 3D reconstruction and selected orthoslices of a twisted Au NR, obtained using 20 μM (*R*)-LipoCYS during chiral overgrowth. Solid white lines indicate the expected crystallographic orientations of {520} facets. White arrows indicate positions where chiral overgrowth occurs. (B) Combined HAADF-STEM tomography (real space) and electron diffraction tomography (reciprocal space) reconstructions of the same particle oriented along [100], [520], [110], [250], and [−250], with rotation angles as indicated. (C) Idealized chiral model of the same nanoparticle, oriented along the same directions as the corresponding panels in (B). All scale bars correspond to 25 nm.

In the simulated absorbance spectra (Figure 2D), two plasmonic bands are found to broaden and red-shift for models resembling the chiral NRs prepared with increasing LipoCYS concentration, again in good agreement with the experimental absorbance spectra. However, a deviation in the agreement is seen for the intensity of the near-IR band in the simulated spectrum of the 90 μM LipoCYS model, which is less intense and more broadened in the experimental spectrum. This is likely due to the idealized nature of our particle models, in contrast with the complex wrinkled geometry in the particles. Further considerations should be made when contrasting simulated and experimental data: ultimately what is represented in simulations is a simplified perspective of the geometry of a single particle, in comparison with a variety of morphological details in a colloid dispersion.

A detailed analysis of the morphology of the produced particles was thus performed using HAADF-STEM tomography for both enantiomers (Figure 3 and Figure S6). Three-dimensional reconstructions of the NPs indicate an evolution from relatively smooth surfaces at a low LipoCYS concentration to densely wrinkled shapes at a high LipoCYS content. Similar morphological studies were also applied to the 90 μM sample (Figure S7). HAADF-STEM showed an average length of the wrinkles of around 22 nm, a distance between them of ∼2 nm, and a thickness of ∼4 nm.

To further investigate the chiral growth mechanism for low LipoCYS concentration, high-resolution HAADF-STEM images were acquired, which indicate that the tips are enclosed by {110} facets (Figure S8). For nanoparticles as complex as those investigated here, 2D HAADF-STEM images do not enable a straightforward identification of the crystallographic nature of the overall surface structure, in particular, the concave surfaces. We therefore performed simultaneous HAADF-STEM and electron diffraction (ED) tomography on a selected NP, obtained using 20 μM (*R*)-LipoCYS (Figure 4 and Movie S1). Orthoslices orthogonal to the [001] direction (Figure 4A) of the HAADF-STEM reconstruction indicate that the particle has a squarelike cross-section, albeit with concave faces. Combination of tomography reconstructions in real and reciprocal space enables a detailed analysis of the various facets in the NP, by orienting it along a given zone axis and inspecting the corresponding part of the NP surface perpendicular to that direction (Figure 4B).

Through the former analysis, we conclude that the observed concave faces mainly consist of two facets belonging to the {520} family. Correspondingly, we present an idealized morphology in Figure S9A. Further inspection of the orthoslices near the NP tips (Figure 4Ai,iii) indicates selective overgrowth at the corners of the two-sided faces (indicated by white arrows), resulting in a seemingly twisted structure. In the case of (*R*)-LipoCYS, this selective overgrowth occurs on the top left and bottom right corners of each concave face, as also illustrated in the idealized model (Figure 4C). The concavity of the NR morphology in these regions can be expected to lead to local {521} facets at the top left and bottom right corners of the lateral facets and {52–1} facets at the top left and bottom left corners. Since these are chiral facets, the mechanism resulting in the final morphology would, therefore, be similar to that described in refs (5 and 21). Finally, an inspection of the ⟨111⟩ and ⟨011⟩ corners (Figure S9B) indicates that they are twisted in a similar manner as the helicoids reported by Lee et al.,^21^ likely intertwined with the presence of chiral facets.

Based on the former morphological studies, it is obvious that at low concentration, LipoCYS follows a mechanism equivalent to that described for standard cysteine, inducing chiral growth upon symmetry breaking of chiral high-Miller-index facets.^5,21^ The resulting morphology is thus similar to the 4-fold twisted gold nanorods described in ref (20). Notwithstanding, whereas cys was used at a concentration of 75 nM, micromolar concentration (20 μM) is required for LipoCYS to induce chirality. We ascribe this difference to the absence of a carboxylate group in LipoCYS, which reduces the affinity for the gold surface. This is in agreement with ref (24), where the *g-*factor was significantly reduced when cysteine was replaced by an analogue containing a methylated carboxylate group.

To elucidate the underlying mechanism governing chiral growth at high LipoCYS concentrations, it is crucial to emphasize that LipoCYS was synthesized with a specific design, aimed at facilitating both interaction with CTAC micelles, through the incorporation of the aliphatic chain, and the gold surface, via the thiol group. Effective interaction with the plasmonic surface becomes apparent at concentrations exceeding 20 μM, as indicated by previous findings. Concerning its impact on the micellar system, the low concentration of LipoCYS utilized in chiral growth experiments, i.e., 0.2% LipoCYS relative to CTAC molarity for the 90 μM sample, is insufficient to induce any discernible alterations in the morphology of CTAC micelles within the bulk phase. This was verified through diffusion-ordered spectroscopy (DOSY) and dynamic light scattering (DLS) experiments, as depicted in Figures S10–S12. On this basis, we hypothesize that a critical threshold exists, at which the local concentration of LipoCYS on the Au surface becomes sufficient to trigger the formation of wormlike micelles over the NP surface. In this scenario, the aliphatic tails of LipoCYS act as anchoring points for the micelles, directing crystal growth into wrinkles. Importantly, analysis of the intermediate samples, i.e., 45 and 75 μM LipoCYS, showed a lower density of wrinkles because their thickness increases up to ∼7 nm (Figure S5). This result can also be elucidated through the earlier hypothesis, given that hydrophobic forces drive LipoCYS toward clustering, thereby initiating the formation of elongated micelles. A lower amount of LipoCYS would thus yield a reduced number of wormlike micelles, leading to a correspondingly lower count of grooves and resulting in the formation of larger wrinkles.

We have contended in this work that chiral growth on gold nanorods can be modulated through rational control over the underlying growth mechanisms, which may provide valuable information for the predictable synthesis of chiral plasmonic nanomaterials. We have demonstrated reproducible control over the obtained nanoparticle structure at different scales: both fine-detailed wrinkles and the larger overall particle geometry can be tailored through the synthesis conditions. Such a variation can be achieved by modifying a single parameter (the concentration of the chiral molecule, LipoCYS) for a given chiral synthesis. These results are supported by observations that the deposition of gold on the nanorod (seed) surface, and even the preference of facet growth, is heavily influenced by the presence of cosurfactants.^21,22,25,26^ It should be noted that above a certain concentration of LipoCYS, the observed chiroptical response decreases significantly as a result of the observed lower degree of order in the wrinkled structure. The relative contribution to the overall chiroptical signature of micelle-directed formation of wrinkles and stabilized facet-directed twisting of the particle geometry warrants further investigation.

An additional relevant finding of this study is related to micellar template growth: to date, this synthetic protocol has been based on applying a very particular type of molecules with axial chirality as inducers of dissymmetry. Herein, we found that molecules with central chirality are also capable of giving rise to the same chiral morphology. Comparison of the chemical structure of LipoCYS and the previously used inducers suggests that micellar template growth requires chiral inducers with two different regions, a polar head with high affinity for the gold surface and a hydrophobic part that endows affinity for the micelles. Taken together, these results suggest an expanded role for cosurfactants that can be applied for the preparation of nanomaterials with chiral morphology. The availability of a diverse range of gold seeds of various shapes and sizes further emphasizes the significance of these findings.

## Supporting Information Available

Electron diffraction (ED) tomography on a selected NP (MPG)
Synthesis and characterizations of both LipoCYS enantiomers and details on preparation, simulations, and microscopy characterization, as well as additional simulations and characterization of chiral gold nanorods (PDF)

## Supplementary Material

nl3c02800_si_001.mpg
