# Supplementary material for: Tuning the Growth of Chiral Gold Nanoparticles Through Rational Design of a Chiral Molecular Inducer
Source: Nano Lett. 2023 Oct 25;23(21):9880–6. doi: 10.1021/acs.nanolett.3c02800 (PMC10636791; doi:10.1021/acs.nanolett.3c02800)
Supplement: Supplementary file 2 — nl3c02800_si_002.pdf [file nl3c02800_si_002.pdf]

SUPPORTING INFORMATION

Tuning the Growth of Chiral Gold Nanoparticles Through Rational Design of a Chiral Molecular Inducer

Kyle Van Gordon,[‡,a] Sandra Baúlde,[‡,b] Mikhail Mychinko,[‡,c] Wouter Heyvaert,[c] Manuel Obelleiro-Liz,[d] Alejandro Criado,[b] Sara Bals,*[,c] Luis M. Liz-Marzán,*[a,e,f,g] Jesús Mosquera*[,b]

[a] CIC biomaGUNE, Basque Research and Technology Alliance (BRTA), 20014 Donostia-San Sebastián, Spain

[b] Universidade da Coruña, CICA – Centro Interdisciplinar de Química e Bioloxía, Rúa as Carballeiras, 15071 A Coruña, Spain

[c] EMAT and NANOlab Center of Excellence, University of Antwerp, B-2020 Antwerp, Belgium

[d] EM3Works, Spin-off of the University of Vigo and the University of Extremadura, PTL Valladares, 36315 Vigo, Spain

[f] Biomedical Networking Research Center, Bioengineering, Biomaterials and Nanomedicine (CIBER-BBN), 20014 Donostia-San Sebastián, Spain

[g] Ikerbasque, 48009 Bilbao, Spain

[h] Cinbio, Universidade de Vigo, 36310 Vigo, Spain

CONTENTS:

Preparation of Chiral Ligands.. S2

Nanoparticle Synthesis... S11

Electron Microscopy.. S13

Computational Modeling.. S14

Supporting Figures.. S15

References... S21

Preparation of chiral ligands

Commercially available N-Boc-(R)-Cys(Trt)-OH, N-Boc-(S)-Cys(Trt)-OH, Decan-1-amine, and triisopropylsilane (TIS) were obtained from BLD pharm. N,N-diisopropylethylamine (DIPEA), tris(2-carboxyethyl)phosphine (TCEP), and trifluoroacetic acid (TFA) were purchased from TCI. O-(7-Azabenzotriazol-1-yl)-N,N,N',N'-tetramethyluronium hexafluorophosphate (HATU) was obtained from Apollo Scientific. Dichloromethane (DCM) was acquired from Scharlau. Deuterated chloroform ($CDCl_3$) and methanol (CD_3OD) were obtained from Deutero GmbH. Analytical thin-layer chromatography was performed using Silicycle aluminum-backed TLC plates. Compounds that were not UV-active were visualized by dipping the plates in a ninhydrin stain. Silica gel flash chromatography was performed using Labkem silica gel (type SGEC-060-500) and HPLC purification was carried out using a Fortis C18 semipreparative column (5 µm, size: 250×10 mm) with Phase A/Phase B gradients (Phase A: H_2O with 0.1% trifluoroacetic acid; Phase B: Acetonitrile with 0.1% trifluoroacetic acid). Proton nuclear magnetic resonance (^1H NMR) and Carbon nuclear magnetic resonance (^{13}C NMR) spectra were measured on Bruker AVANCE III HD 400 Nuclear Magnetic Resonance spectrometer and were referenced relating to residual proton resonances in $CDCl_3$ (at d 7.24 ppm and 77.23 ppm) and CD_3OD (at d 4.87 or 3.31 ppm and 49.15 ppm). ^1H NMR splitting patterns are assigned as singlet (s), doublet (d), triplet (t) or quartet (q). Splitting patterns that could not be readily interpreted are designated as multiplet (m).

Synthesis of LipoCYS

Both enantiomers of the **LipoCYS** molecule were synthesized following a procedure reported by Neal K. Devaraj et al.[1]

Synthesis of N-Boc-(*R*)-Cys(Trt)-Decan

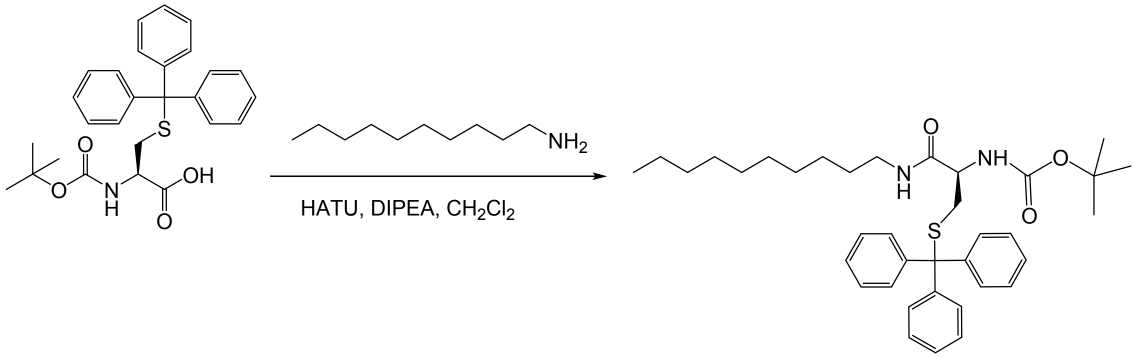

A solution of **N-Boc-(*R*)-Cys(Trt)-OH** (1 g, 2.16 mmol) in CH_2Cl_2 (14.5 mL) was stirred at 0 ºC for 10 min, and then HATU (820 mg, 2.16 mmol) and DIPEA (376 µL, 2.16 mmol) were successively added. After 10 min stirring at 0 ºC, decan-1-amine (339 mg, 2.16 mmol) was added. After 1 h stirring at room temperature, the reaction mixture was washed with $NaHCO_3$(sat) (3 × 10 mL). The organic layer was dried (Na_2SO_4), filtered and concentrated, providing a yellow oil, which was purified by flash chromatography (0–3% MeOH in CH_2Cl_2), affording the expected product as a yellow oil [96%, Rf = 0.52 (1% MeOH in CH_2Cl_2)].

NMR Spectra of N-Boc-(*R*)-Cys(Trt)-Decan

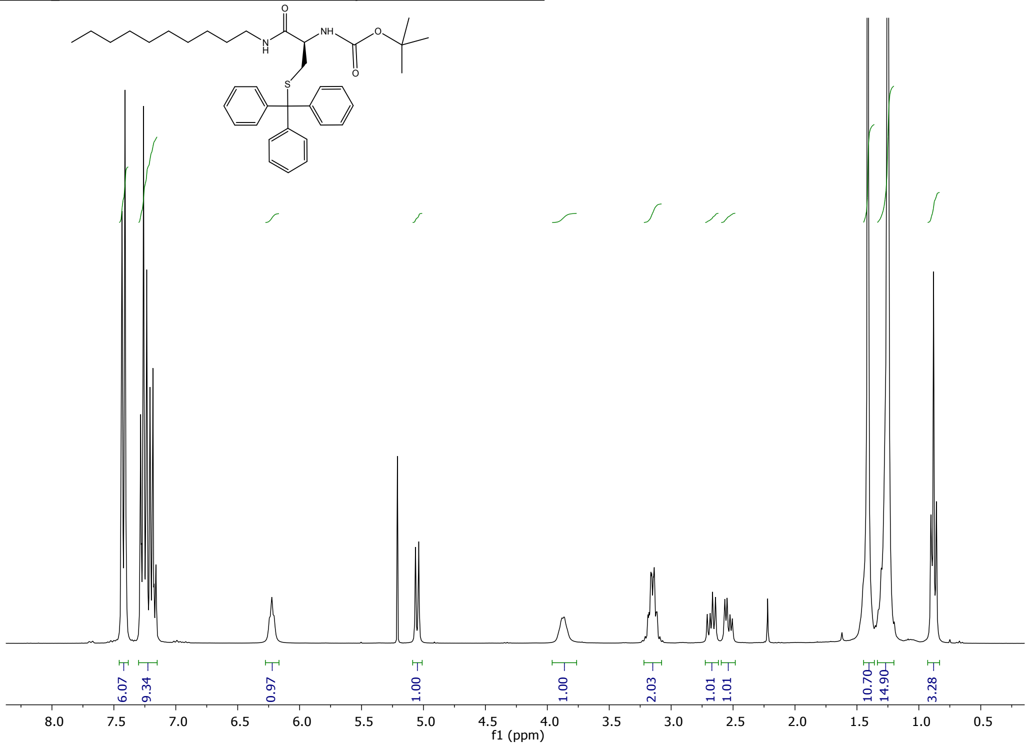

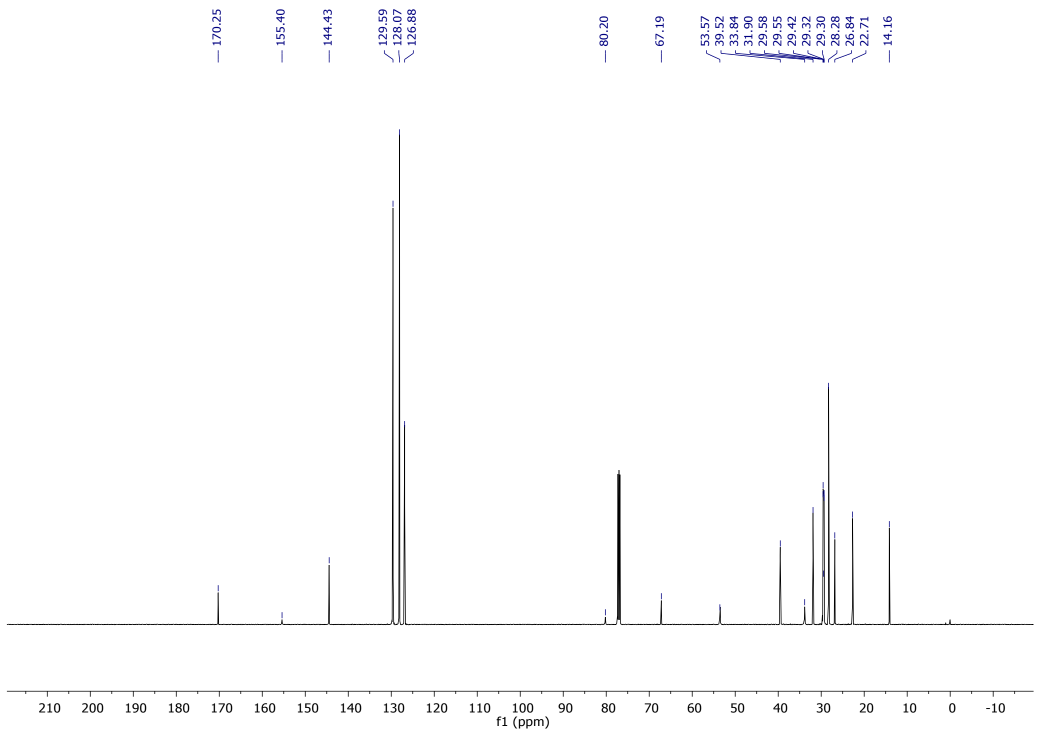

^{1}H NMR (300 MHz, Chloroform-*d*) δ: 7.46 – 7.36 (m, 6H), 7.30 – 7.14 (m, 9H), 6.22 (t, *J* = 5.8 Hz, 1H), 5.05 (d, *J* = 7.9 Hz, 1H), 3.87 (m, 1H), 3.19 – 3.09 (m, 2H), 2.73-2.62 (dd, J= 12.7, 7.1 Hz, 1H), 2.60-2.48 (dd, J= 12.7, 5.6 Hz, 1H), 1.39 (s, 9H, 3 × CH_3), 1.23 (s, 14H), 0.91(t, 3H) ppm. ^{13}C NMR (75 MHz, $CDCl_3$): δ 170.25(1C), 155.40(1C), 144.43(3C), 129.59(6CH), 128.07(6CH), 126.88(3CH), 80.20(1C), 67.19(1C), 53.57(1CH), 39.52(1CH_2), 33.84(1CH_2), 31.90(1CH_2), 29.58(1CH_2), 29.55(1CH_2), 29.42(1CH_2), 29.32(1CH_2), 29.30(1CH_2), 28.28 (3CH_3), 26.84(1CH_2), 22.71(1CH_2), 14.16 (1CH_3) ppm.

Synthesis of N-Boc-(*S*)-Cys(Trt)-Decan

The (*S*) enantiomer was prepared following the same procedure, but using **N-Boc-(*S*)-Cys(Trt)-OH** as a precursor.

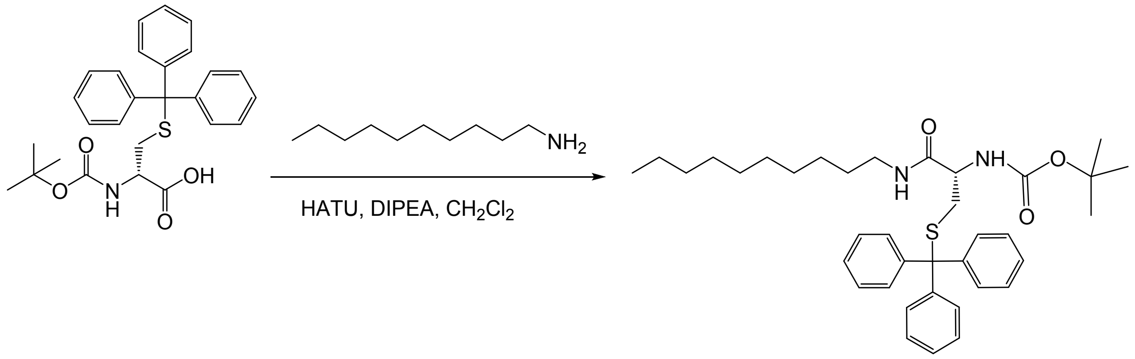

NMR Spectra of N-Boc-(S)-Cys(Trt)-Decan

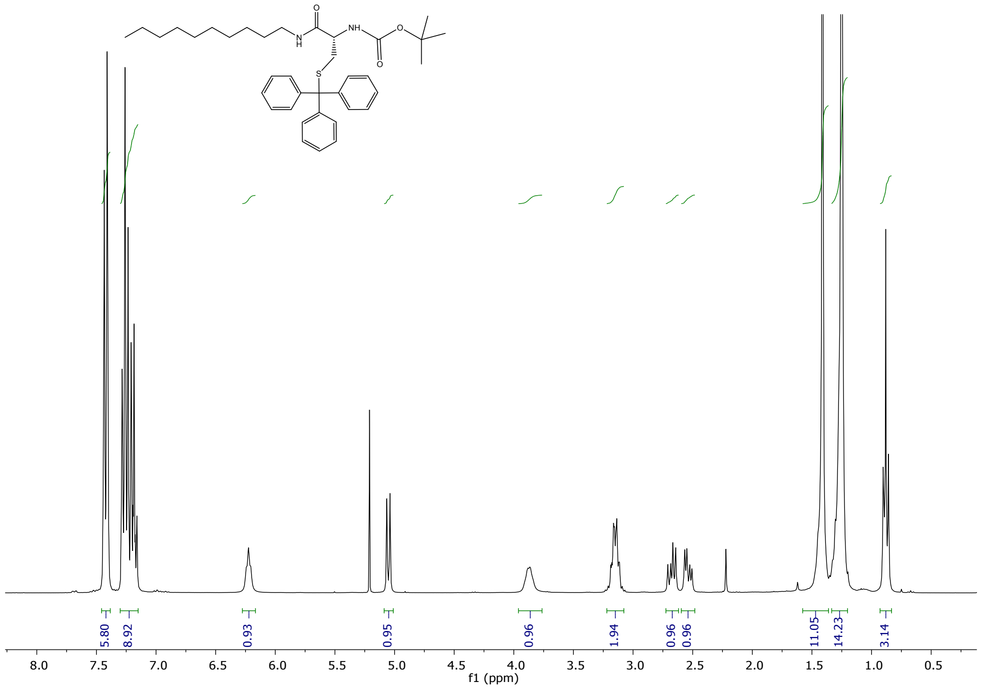

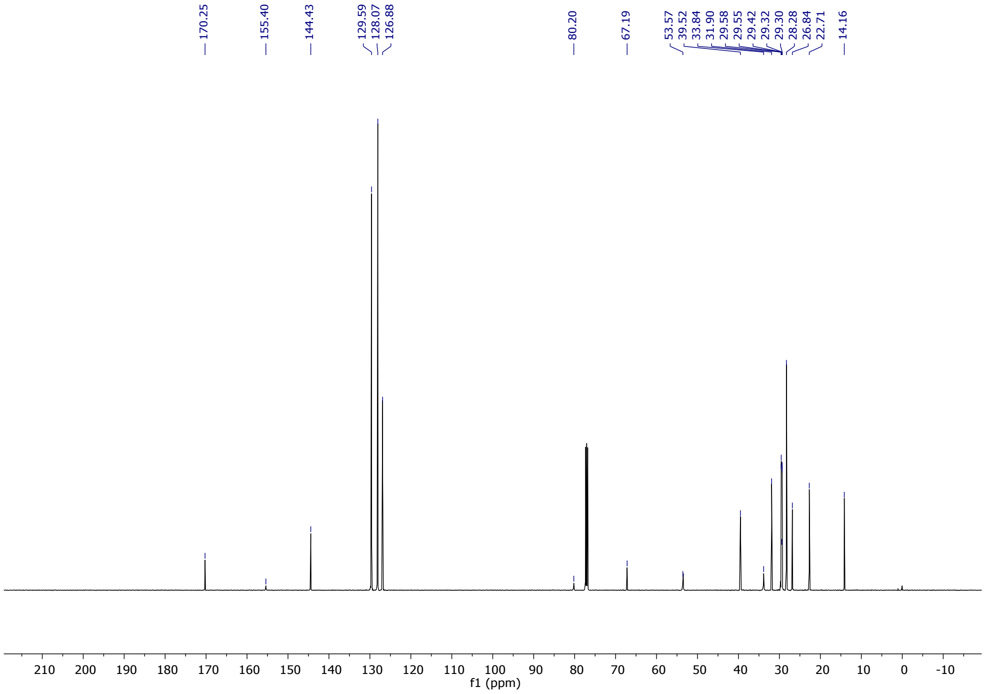

^{1}H NMR (400 MHz, Chloroform-*d*): δ 7.48 – 7.36 (m, 6H), 7.32 – 7.12 (m, 9H), 6.23 (t, J = 5.8 Hz, 1H), 5.05 (d, J = 7.9 Hz, 1H), 3.87 (m, 1H), 3.15 (m, 2H), 2.74-2.60 (dd, J = 12.7, 7.1 Hz, 1H), 2.60-2.48 (dd, J = 12.7, 5.6 Hz, 1H), 1.41 (s, 9H, 3 × CH_3), 1.25 (s, 14H), 0.88(m, 3Hppm. ^{13}C NMR (100 MHz, $CDCl_3$): δ 170.25(1C), 155.40(1C), 144.43(3C), 129.59(6CH), 128.07(6CH), 126.88(3CH), 80.20(1C), 67.19(1C), 53.57(1CH), 39.52(1CH_2), 33.84(1CH_2), 31.90(1CH_2), 29.58(1CH_2), 29.55(1CH_2), 29.42(1CH_2), 29.32(1CH_2), 29.30(1CH_2), 28.28 (3CH_3), 26.84(1CH_2), 22.71(1CH_2), 14.16 (1CH_3) ppm.

Synthesis of (*R*)-2-amino-N-decyl-3-mercaptopropanamide, (*R*)-LipoCYS

The synthesis of the (*R*) enantiomer of **LipoCYS** is described below.

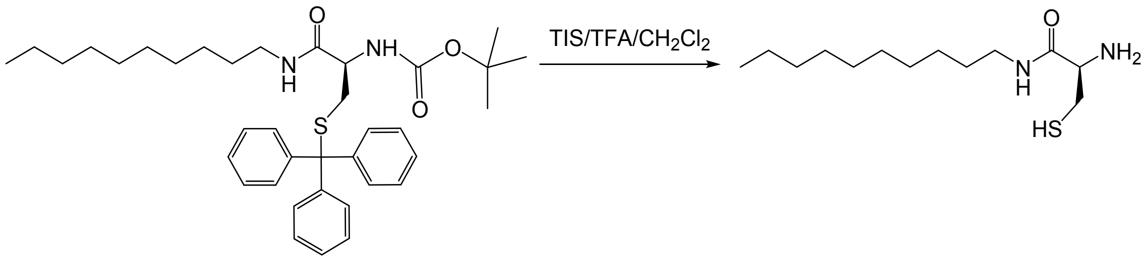

N-Boc-(*R*)-Cys(Trt)-Decan (100 mg) was dissolved in 2 mL of TFA/CH_2Cl_2/TIS (225:225:50) and the mixture was stirred at room temperature for 30 min. After removal of the solvent, the residue was dried under high vacuum for 1 h. Then, the corresponding residue was diluted in milli-Q H_2O containing 1 mM of TCEP, filtered using a 0.2 µm syringe-driven filter, and the crude solution was purified by HPLC, yielding the purified compound as a colorless oil [R_T= 18 min (C18 semipreparative column, 70% Phase A in Phase B, 30 min, and then 5% Phase A in Phase B, 10 min)].

NMR Spectra of (*R*)-LipoCYS

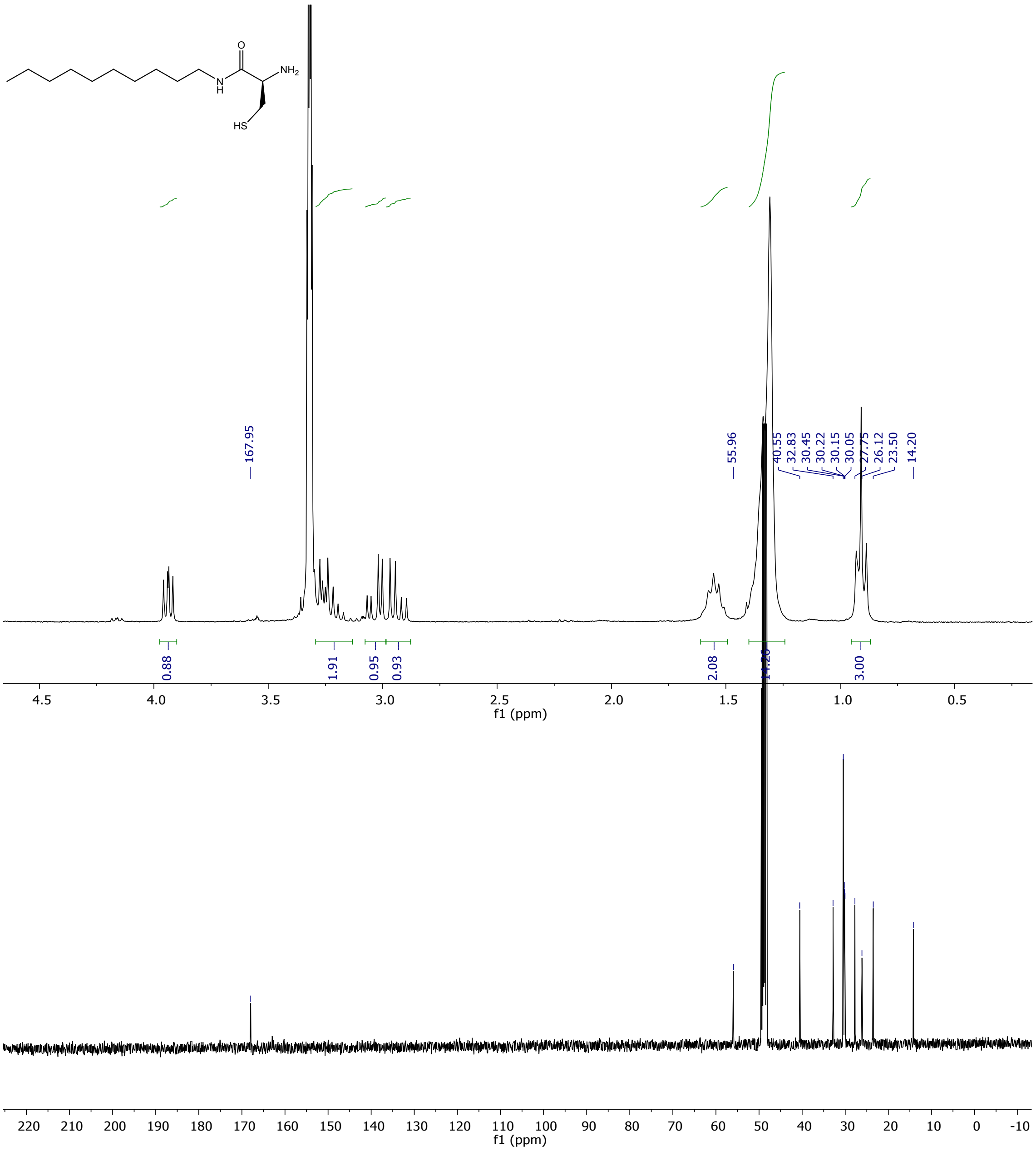

^{1}H NMR (400 MHz, MeOD) δ 3,97 (dd, J=5.22, 6.88 Hz, 1H), 3.34-3.20 (m, 2H), 3.08-2.98 (dd, J=14.59, 6.88 Hz, 1H) 2.98-2.88 (dd, J=14.59, 5.22 Hz, 1H), 1.61-1.53 (m, 2H), 1.40-1.25 (m, 14H), 0.91 (t, J=6.9 Hz, 3H). ^{13}C NMR (100 MHz, MeOD): δ 167.95 (1C), 55.96 (1CH), 40.55 (1CH_2), 32.83 (1CH_2), 30.45 (2CH_2), 30.22 (1CH_2), 30.15 (1CH_2), 30.05 (1CH_2), 27.75 (1CH_2), 26.12 (1CH_2), 23.50 (1CH_2), 14.20 (1CH_3). HRMS (ESI+): *m/z* calculated for $C_{13}H_{27}N_2O_S[M+H]^+$ 261.200, found 261.199.

Synthesis of (*S*)-2-amino-N-decyl-3-mercaptopropanamide, (*S*)-LipoCYS

The (*S*) enantiomer was prepared following the same procedure, but using N-Boc-(S)-Cys(Trt)-Decan as a precursor.

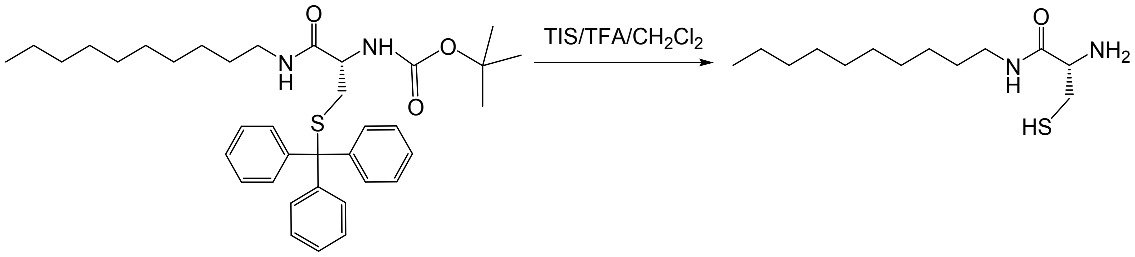

NMR Spectra of (*S*)-LipoCYS

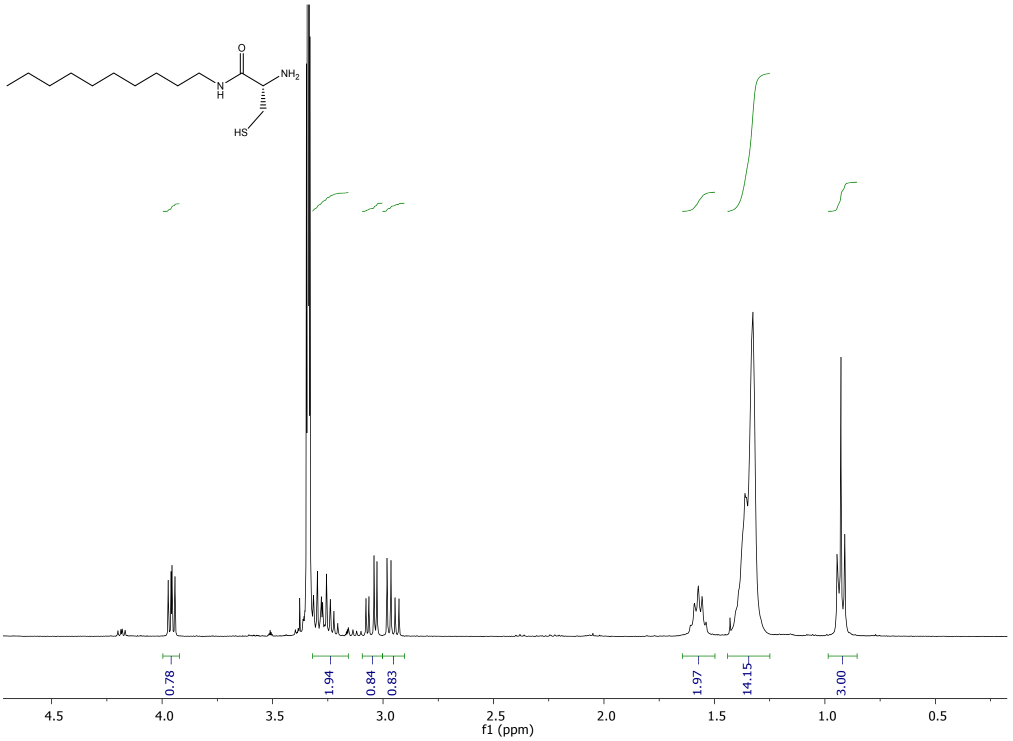

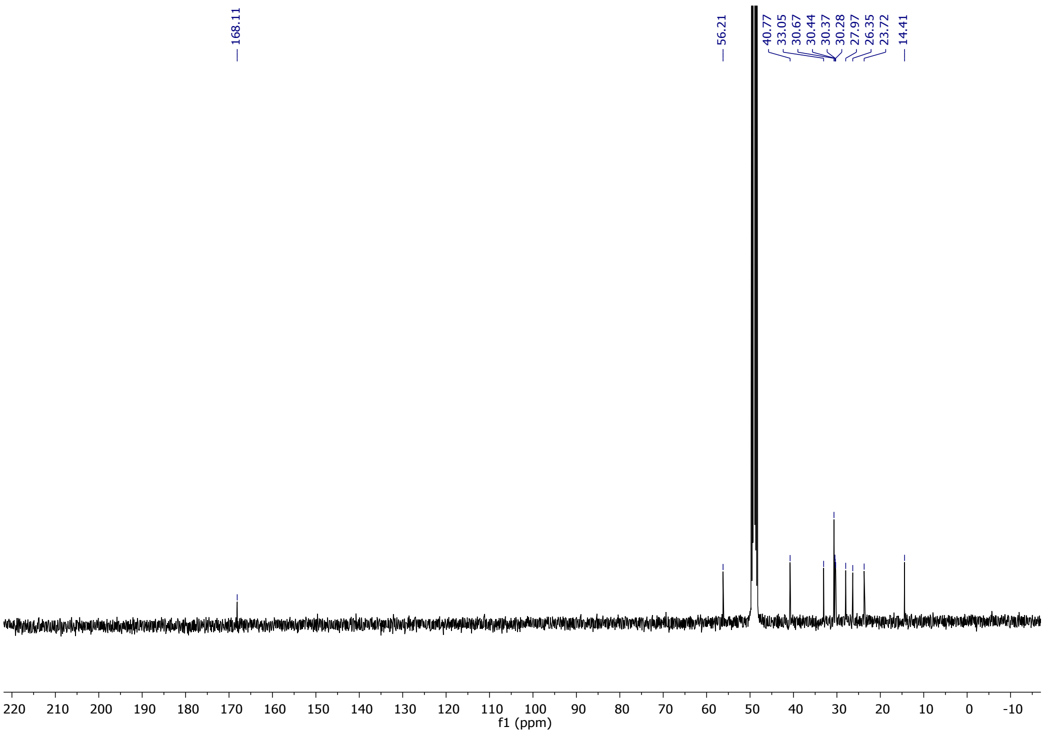

^{1}H NMR (400 MHz, MeOD) δ: 3,97 (dd, J=5.22, 6.90 Hz, 1H), 3.34-3.17 (m, 2H), 3.09-3.00 (dd, J=14.59, 6.90 Hz, 1H), 3.00-2.91 (dd, J=14.59, 5.22 Hz, 1H) 1.62-1.50(m, 2H), 1.44-1.25 (m, 14H), 0.93 (t, J=6.9 Hz, 3H) ppm. ^{13}C NMR (100 MHz, $CDCl_3$) δ:168.11(1C), 56.21(1CH), 40.77(1CH_2), 33.05(1CH_2), 30.67(2CH_2), 30.44(1CH_2), 30.37(1CH_2), 30.28(1CH_2), 27.97(1CH_2), 26.35(1CH_2), 23.72(1CH_2), 14.41(1CH_3) ppm. HRMS (ESI+): *m/z* calculated for $C_{13}H_{27}N_2O_S$[M+H]$^+$ 261.200, found 261.199.

Nanoparticle synthesis

Chemicals

Cetyltrimethylammonium bromide (CTAB; 99%) and cetyltrimethylammonium chloride (CTAC; 99%) were purchased from ThermoFisher. Silver nitrate ($AgNO_3$; ≥ 99.0%), n-decanol (for synthesis; ≥ 99.0%), sodium borohydride ($NaBH_4$; 99%), and ascorbic acid (AA; ≥ 99%) were purchased from Sigma-Aldrich. Hydrogen tetrachloroaurate(III) hydrate ($HAuCl_4$; 99.9%) was sourced from AlfaAesar, and hydrochloric acid (HCl; ACS, ISO grade, 37% w/v) was sourced from Scharlau.

Gold seeds for preparation of gold mini-rods

To a glass vial, 5 mL of stock solution #1 (50 mM CTAB, 13.5 mM n-decanol) was transferred before addition of 50 µL $HAuCl_4$ solution (aq., 50 mM) under constant magnetic stirring. After thorough sonication, 25 µL AA solution (aq., 100 mM) was added under constant magnetic stirring (200 rpm) until the solution became colorless. The stirring was then increased to 1500 rpm, and a 200 µL aliquot of $NaBH_4$ solution (aq., 20 mM) was quickly added by rapidly depressing the plunger of the micropipettor; the solution turned a deep brown color. Gold seeds were prepared in triplicate, and UV-Vis spectra were analyzed for reproducibility and target peaks (350 and 480 nm) indicate the formation of Au seeds of around 1.5 nm, as described in the literature.[2] Au seeds were rested at room temperature for at least one hour, but not more than four hours, prior to further use.

Gold mini-rod seeds for large gold nanorod (AuNR) preparations

In a glass vial, 80 mL of stock solution #1, 640 µL of $AgNO_3$ solution (aq., 10 mM), and 4.4 mL of HCl solution (aq., 1 M) were mixed before 800 µL $HAuCl_4$ solution (aq., 50 mM) was added under constant magnetic stirring. After thorough sonication, 1040 µL AA solution (aq., 100 mM) was added under constant magnetic stirring (200 rpm) until the solution became colorless. The stirring was then increased to 600 rpm, and 4.8 mL of Au seed dispersion was added; after an overnight incubation at 25 °C, the solution turned a brownish color. UV-Vis spectra were analyzed for a target LSPR peak of 734 nm, which corresponds to particle dimensions of ca. 21 nm × 7.5 nm. Initial centrifugation at 13000 rpm for 30 minutes was used to remove larger particles; the supernatant was then subjected to three additional rounds of centrifugation at 14500 rpm for 60 minutes. After

each round, the supernatant was discarded and the particles resuspended in 10 mM aqueous CTAB solution.

Gold nanorods (AuNRs) for chiral preparations

In a glass vial, 80 mL of stock solution #2 (50 mM CTAB, 11 mM n-decanol), 9.6 mL HCl solution (aq., 1 M), and 1.2 mL $AgNO_3$ solution (aq., 50 mM) were mixed before 800 μL $HAuCl_4$ solution (aq., 50 mM) was added under constant magnetic stirring. After thorough sonication, 640 μL ascorbic acid solution (aq., 100 mM) was added under constant magnetic stirring (200 rpm) until the solution became colorless. The stirring was then increased to 600 rpm, and 7 μL of cleaned gold mini-rod seeds ($[Au^0]$ = 29.8 mM) was added; after an overnight incubation at 16 °C in a temperature-controlled water bath, the solution turned a reddish-brown color. Synthesized nanorods were purified by three rounds of centrifugation (7500 rpm, 30 min); after each round, the supernatant was discarded, and the particles redispersed in 1 mM CTAC. To assist removal of CTAB and Ag^+ ions, nanorod dispersions were heated at 60 °C for 30 min before each round of centrifugation.

Use of large gold nanorod seeds for chiral preparations

Chiral preparations were made with two different batches of Au NR seeds (**Figure S1**). Regardless of the used batch, $[Au^0]$ was kept constant for all chiral preparations (20.8 μM). Batch 1 (142.0 ± 10.2 × 31.6 ± 2.11 nm) was used for chiral preparations using the (*R*) enantiomer of LipoCYS; Batch 2 (141.6 (± 10.1) × 26.7 (± 1.6) nm) was used for chiral preparations using the (*S*) enantiomer of LipoCYS.

Chiral gold nanorods

Chiral Au NRs were prepared in 2 mL reaction volumes at 40 °C; CTAC and AA concentrations were kept constant at 44 mM and 700 mM, respectively, and $[Au^{3+}]$:$[Au^0]$ was kept constant at 8.9 (185 μM and 20.8 μM, respectively). For all chiral preparations, and after a 30 min incubation, chiral nanorods were spun down (3250 rpm, 10 min) and resuspended in Milli-Q water. This process was repeated twice to remove excess AA and CTAC from solution.

Estimation of relative LipoCYS concentration

Considering the average dimensions of the Au NR seeds (142 nm x 31.6 nm) and assuming a cylindrical shape, the surface area per NR would be SA = 15665 nm^2 and the volume per NR V = 111366 nm^3. Considering the initial Au NR seed concentration of 2.08×10^{-5} mol/L, this would translate into 1.90476×10^{12} Au NR/L, with a total SA_T = 6.0 × 1013 nm 2, in 2 mL of dispersion.

For each LipoCYS molar concentration, the number of molecules can be readily calculated as: LipoCYS molecules = [LipoCYS] (mol/L) ×vol. dispersion (L) x N_A (molecules / mol), where N_A is the Avogadro number. Again, for 2 mL:
20 µM ⇔ 2.4×10^{16} LipoCYS molecules
45 µM ⇔ 5.4×10^{16} LipoCYS molecules
75 µM ⇔ 9.0×10^{16} LipoCYS molecules
90 µM ⇔ 1.1×10^{17} LipoCYS molecules
200 µM ⇔ 2.4×10^{17} LipoCYS molecules

As a result, the relative number of available LipoCYS molecules per nm^2 Au NR surface can be estimated as:
20 µM ⇔ 404
45 µM ⇔ 908
75 µM ⇔ 1514
90 µM ⇔ 1816
200 µM ⇔ 4036

Finally, the relative number of CTAC molecules (44 mM) per LipoCYS molecule would be:
20 µM ⇔ 2200
45 µM ⇔ 978
75 µM ⇔ 587
90 µM ⇔ 489
200 µM ⇔ 220

Electron Microscopy

Electron tomography

High resolution scanning transmission electron microscopy (STEM) and electron tomography experiments were performed using a “cubed” Thermo Fisher Scientific Themis Z instrument operated at 300 kV in HAADF-STEM mode. High angle annular dark field STEM (HAADF-STEM) tomography series were acquired over an angular range of ±75°, with a tilt increment of 3°, and were used as an input for SIRT reconstruction using the Astra Toolbox 1.8 for MATLAB R2019A.[3] Electron diffraction tomography (ED) series was acquired over an angular range of ±75°, with a continuous tilting (~0.6°/s) and were reconstructed using the PETS2.0 software. Visualization of the 3D reconstructions was performed using the Amira 5.4.0 software.

Surface helicity analysis

The helical morphology of chiral AuNRs was quantitatively characterized using an updated version of our previously reported method.[4] First, the surface of an Au NR was extracted by segmentation of the electron tomography reconstruction, by manually thresholding the reconstruction followed by denoising through several consecutive morphological opening and closing operations. Next, the binarized data were converted to a triangulated mesh using the Thermofisher Amira 5.4.0 software. The center of mass of the resulting mesh was selected as the origin and the helical axis was determined by principal component analysis (PCA) applied to the coordinates of the vertices in the mesh. The principal component corresponds to the long axis of the NR and selected as the helical axis. Next, the normal vector of each surface element was used to determine the helical inclination angle of each surface element with respect to the helical axis. Subsequently, the helicity function $H(\alpha,\rho)$ and total helicity H_{tot}, as described in ref. 4, were calculated by integrating over the surface of the NR:

$$H(\alpha,\rho) := \frac{\iint_S \text{sign}(\alpha')\ \delta(\alpha - |\alpha'|)\ \delta(\rho - \rho')\ dS}{\iint_S dS}$$

$$H_{tot} := \frac{\iint_S \text{sign}(\alpha')\ dS}{\iint_S dS}$$

Here, α' and ρ' are the inclination angle and radius of each point on the surface S, respectively. Since positive inclination angles correspond to right-handed helicity and negative inclination angles to left-handed helicity, the total helicity H_{tot} is a scalar value between -1 (perfect left-handed helicity) and +1 (perfect right-handed helicity). Similarly, the helicity function $H(\alpha,\rho)$ is a distribution indicating which handedness is prevailing at each radius from the helical axis, for each inclination angle, thus giving insights into the helical structure of a NR.

Computational Modeling

Numerical Solution of Maxwell's Equations: The M3 solver[5-7] was used to perform full-wave electromagnetic simulations. The full-wave solutions are based on surface-integral equations (SIEs) discretized by the method of moments (MoM). In SIE-MoM, the parametrization and subsequent numerical analysis are both restricted to the two-dimensional boundary surfaces of the particles, rather than a 3D space embedding of the material structure, which results in a drastic reduction in the number of unknowns

compared with other approaches. SIE-MoM is robust against instabilities produced by rapid spatial variations of the permittivity, as is usually the case in plasmonic structures. Gold was described through its frequency-dependent complex permittivity, taken from optical measurements.[8,9]

Nanoparticle Models: The nanorod models were designed to resemble the experimental 3D tomographic reconstructions of NPs obtained using 20, 45 and 90 μM LipoCYS. The models consist of a twisted nanorod core with square section (70 nm × 70 nm) and 170 nm in length, on which 16 or 25 helical grooves are excavated all around (for the second and third models, respectively), giving rise to 6 or 16 helical wrinkles that unfold along the lateral surfaces. The helices have two leveled and two inclined steps per pitch, with tilt angles of 60 º and 30 º in the inclined steps, respectively. The corresponding wrinkle widths are 8 nm and 4 nm, and the separation distances between wrinkles are 12 nm and 7 nm, respectively. Groove depths are 6 nm and 13 nm, respectively. The modeling and meshing of the models were performed with Blender 3.3, Solidworks and Hypermesh respectively. Having a high-quality, clean mesh, with a consistent aspect ratio across all elements is crucial for the results of the simulation. Mesh quality is key in obtaining accurate results and for the problem to be solved efficiently in terms of time and resources, and with fidelity to the underlying physics. Illumination was based on left- (right-) circularly polarized plane waves impinging from multiple directions. Overall responses were calculated by averaging over light incidence angles.

Supporting Figures

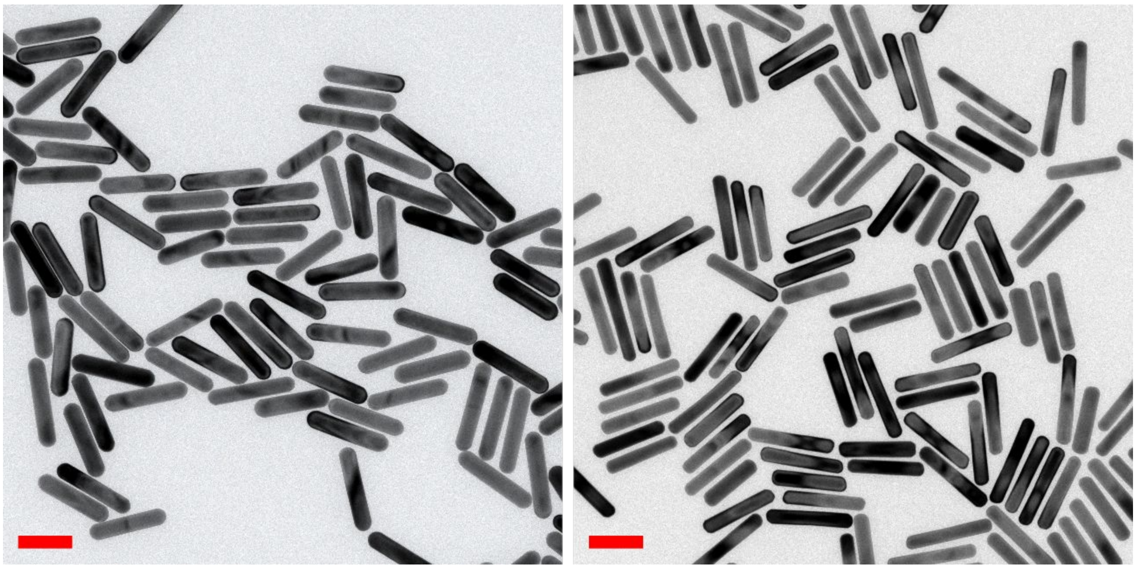

Figure S1. Representative TEM images of AuNR seeds (left = Batch 1, right = Batch 2) for chiral preparations. Scale bars = 100 nm.

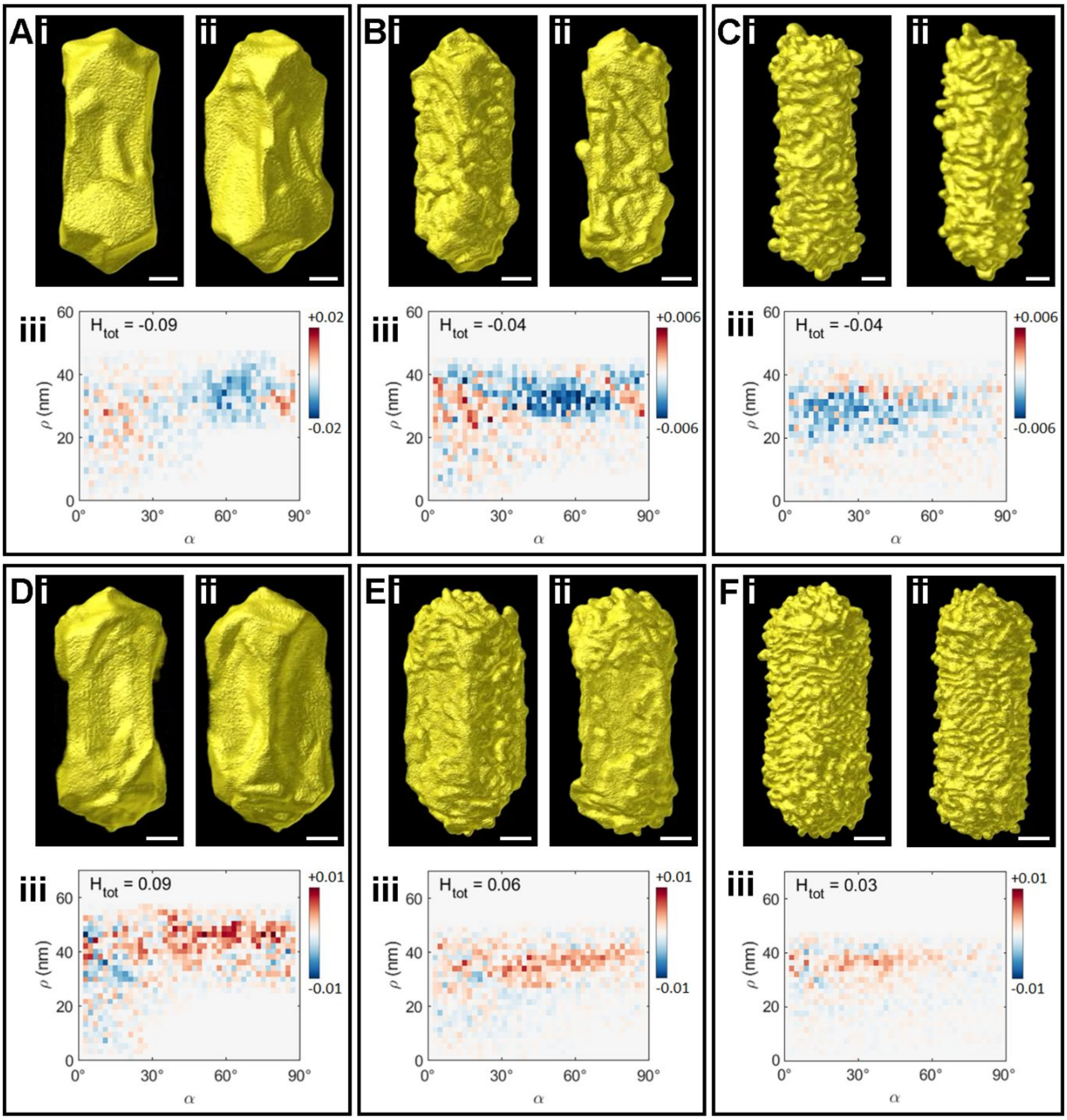

Figure S2. Isosurface visualizations of the 3D reconstructions (i, ii) for Au NRs obtained using different concentrations of (*S*)-LipoCYS (top) and (*R*)-LipoCYS (bottom) (A,D: 20 μM; B,E: 45 μM; C,F: 90 μM). Presented images are made along different viewing angles (oriented 45° relatively to each other) for each particle. Plots of the corresponding helicity function (iii; red: right-handed; blue: left-handed) are provided for each 3D reconstruction. Technical details on how to compute these helicity functions are provided in the "Electron Microscopy" section (page S14). The Au NRs obtained by (*S*)-LipoCYS are found to yield a left-handed helicity (blue), whereas those obtained by (*R*)-LipoCYS are right-handed. All scale bars are 25 nm.

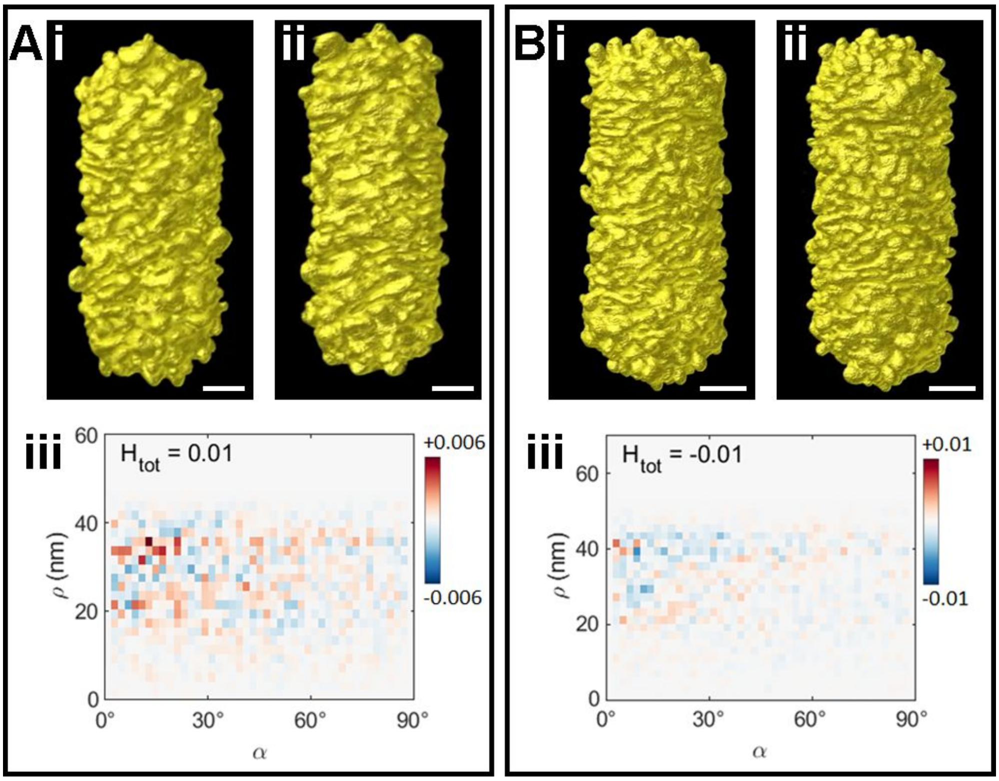

Figure S3. Isosurface visualizations of the 3D reconstructions (i,ii) for Au NRs obtained using 200 µM of (S)-LipoCYS (A) and (R)-LipoCYS (B). Presented images are made along different viewing angles (oriented 45° relatively to each other) for each particle. (iii) Plots of the corresponding helicity function are provided for each 3D reconstruction. Technical details on how to compute these helicity functions are provided in the "Electron Microscopy" section. A less defined helicity plot was obtained, which is reflected in the equal appearance of red and blue features in both panels iii, indicating undefined chirality.

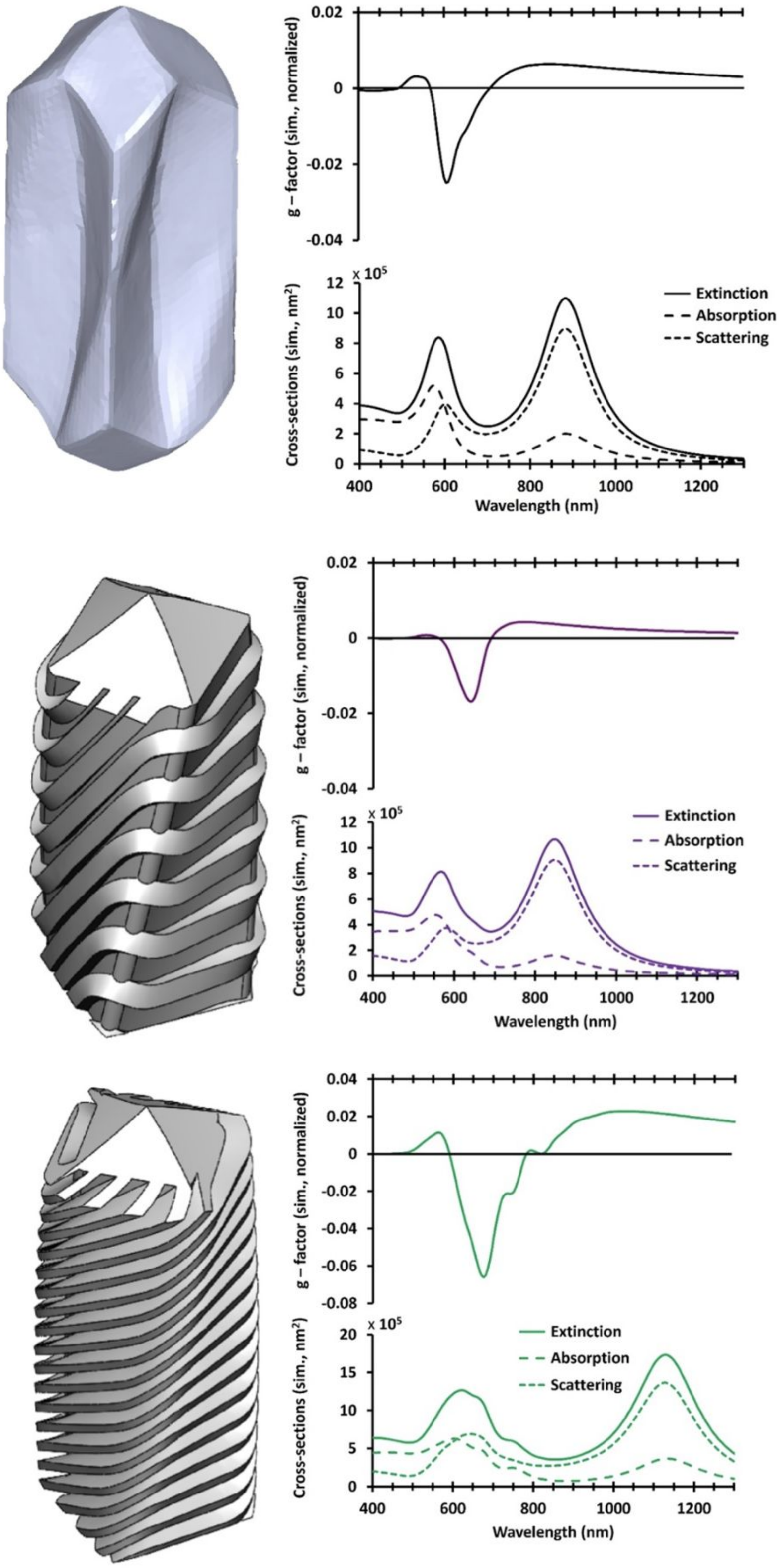

Figure S4. Electromagnetic simulations for 3D models (SolidWorks/HyperMesh) of chiral nanorods synthesized with 20 (top), 45 (middle) and 90 μM (bottom) (*R*)-LipoCYS. For each model: simulated *g*-factor spectra and cross-section spectra for a randomly oriented distribution of nanorods, under illumination with left- and right-handed circularly polarized light (cross-section spectra are shown as an average for left- and right-circular polarizations).

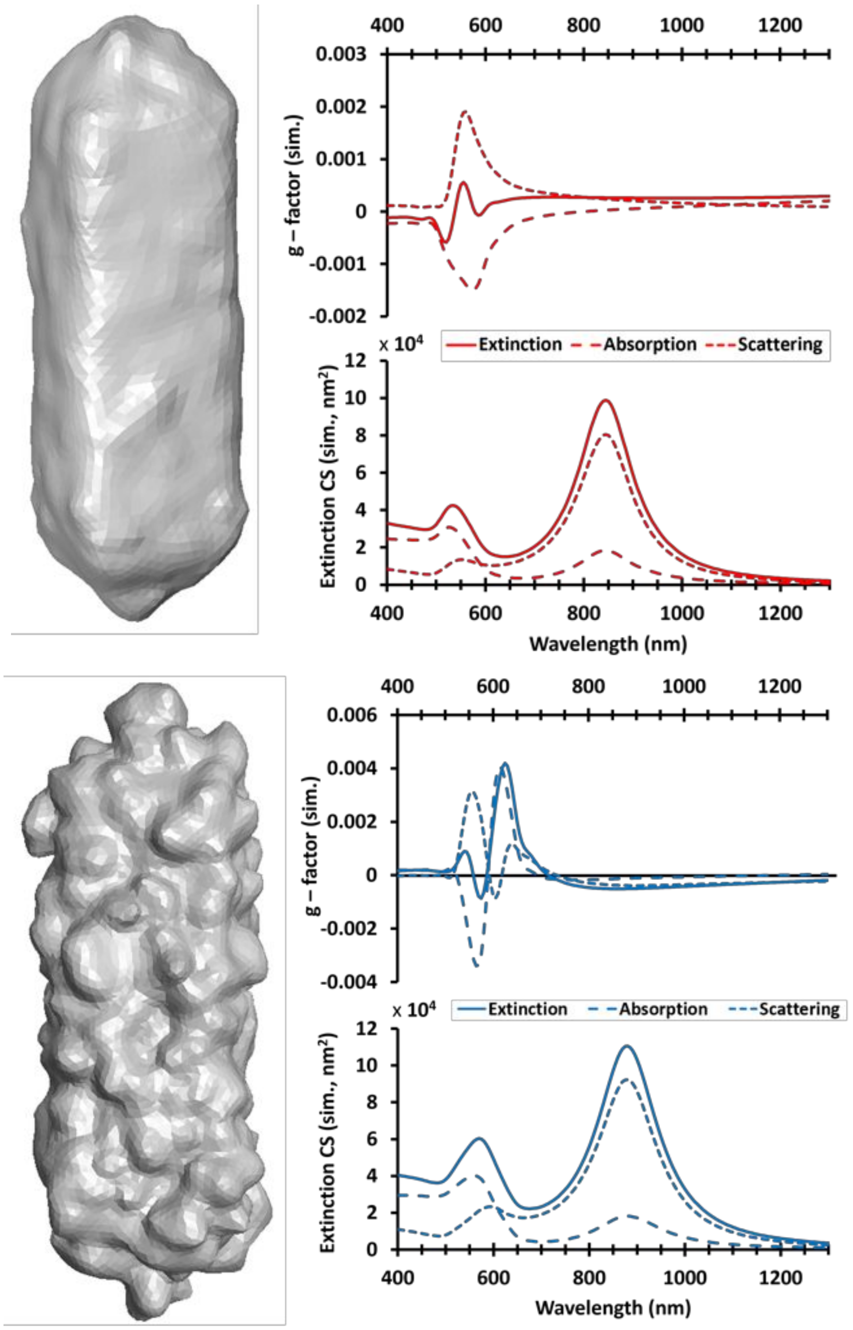

Figure S5. Electromagnetic simulations for a 3D model of Au NRs with disordered surface features, resulting in strongly decreased *g*-factor. Spectra and cross-section spectra are shown for randomly oriented distributions of nanorods, under illumination with left- and right-handed circularly polarized light (cross-section spectra are shown as an average for left- and right- circular polarizations).

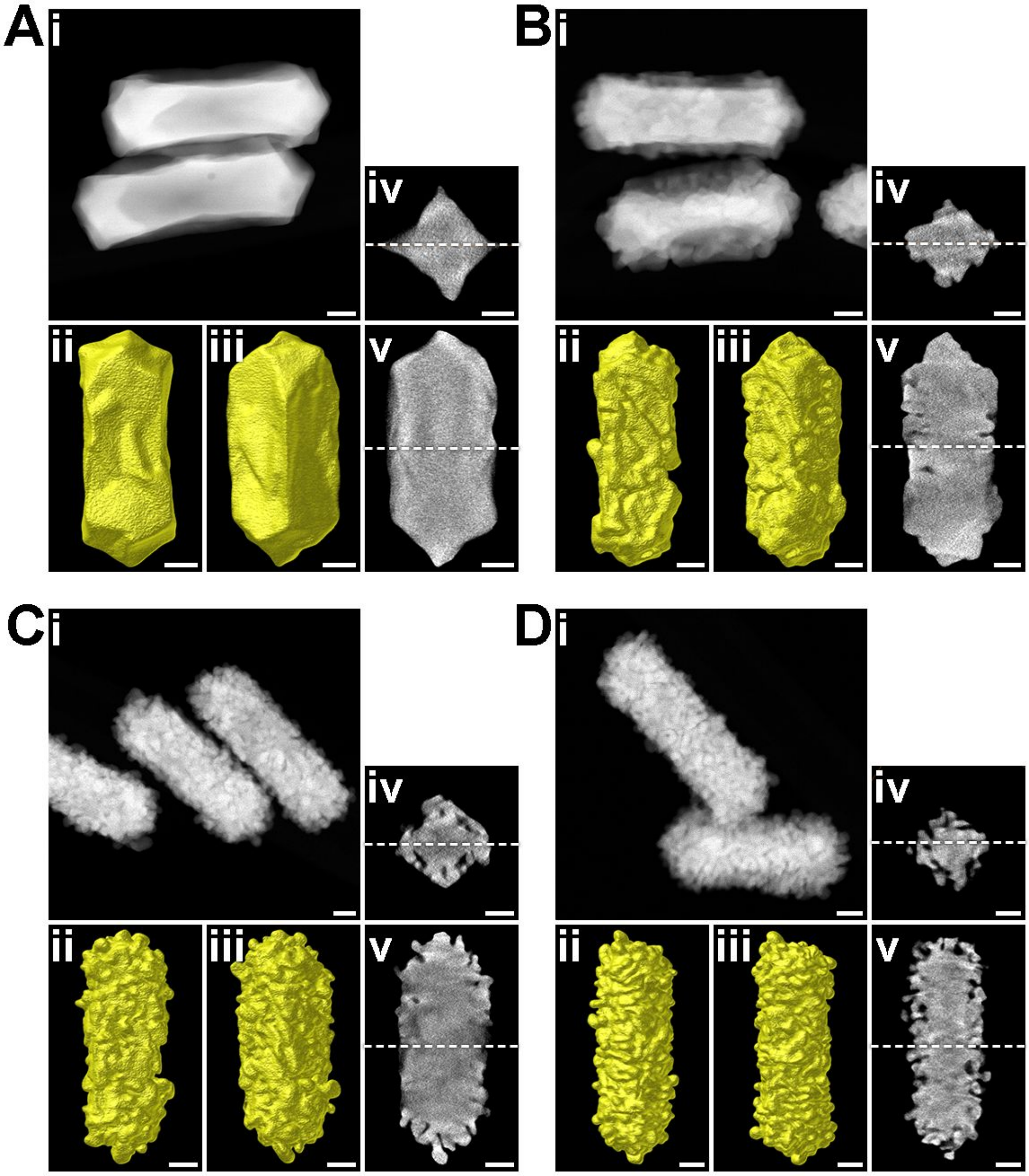

Figure S6. Morphological characterization of Au NPs obtained by increasing the concentration of (*S*)-LipoCYS (A: 20 μM, B: 45 μM, C: 75 μM, D: 90 μM) during chiral overgrowth. The morphological characterization for each sample includes: (i) HAADF-STEM image of several representative nanoparticles; (ii, iii) Visualizations of the 3D reconstructions presented along different viewing angles (oriented 45° relative to each other); (iv, v) selected orthoslices extracted from the 3D reconstructions, perpendicular to the longitudinal and transverse axes, at the center of the NRs. White dashed lines represent the relative positions of slices shown in (iv) and (v). All scale bars are 25 nm.

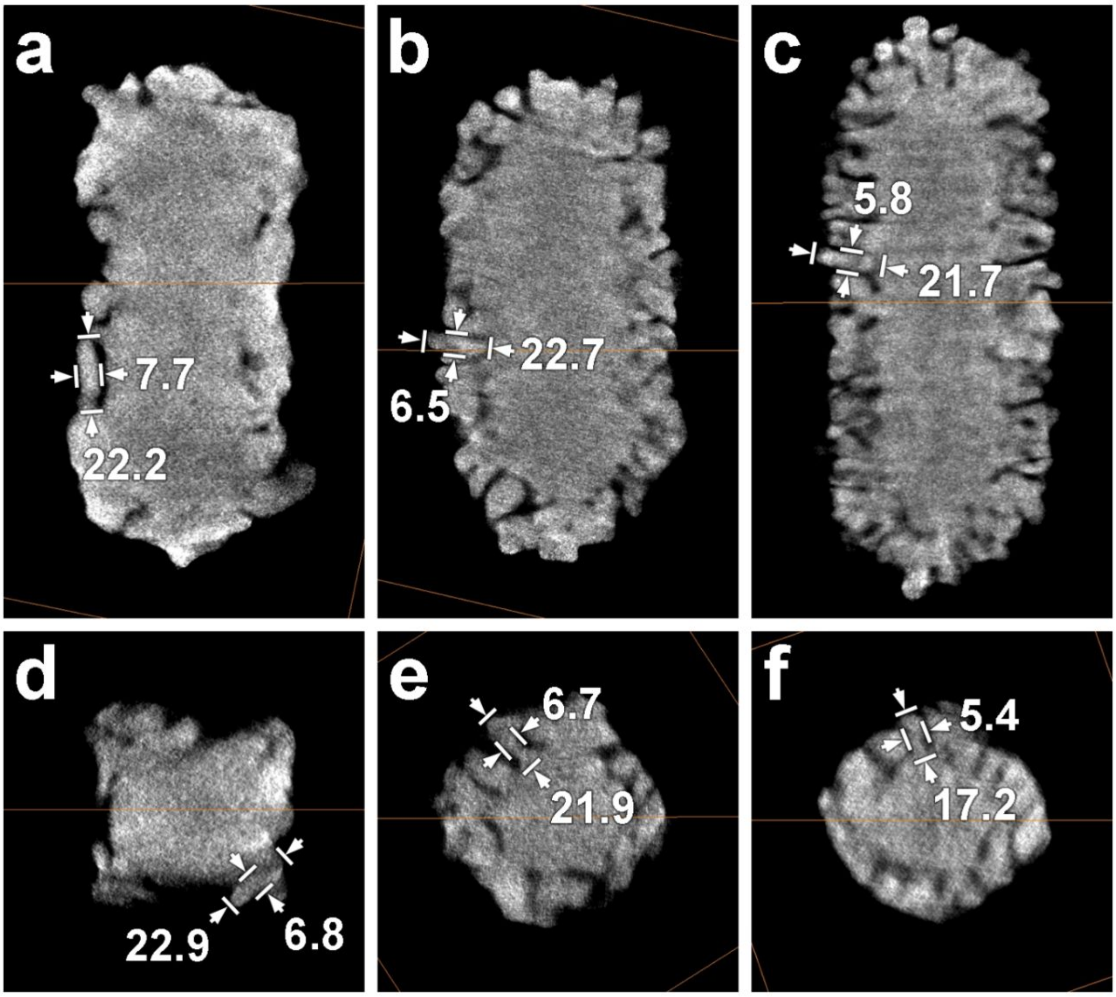

Figure S7. Selected orthoslices extracted from the 3D reconstructions, perpendicular to the longitudinal and transverse axes, at the center of chiral Au NRs prepared with different concentrations of of (*R*)-LipoCYS (a,d: 45 μM, b,e: 75 μM, c,f: 90 μM). All values are given in nm.

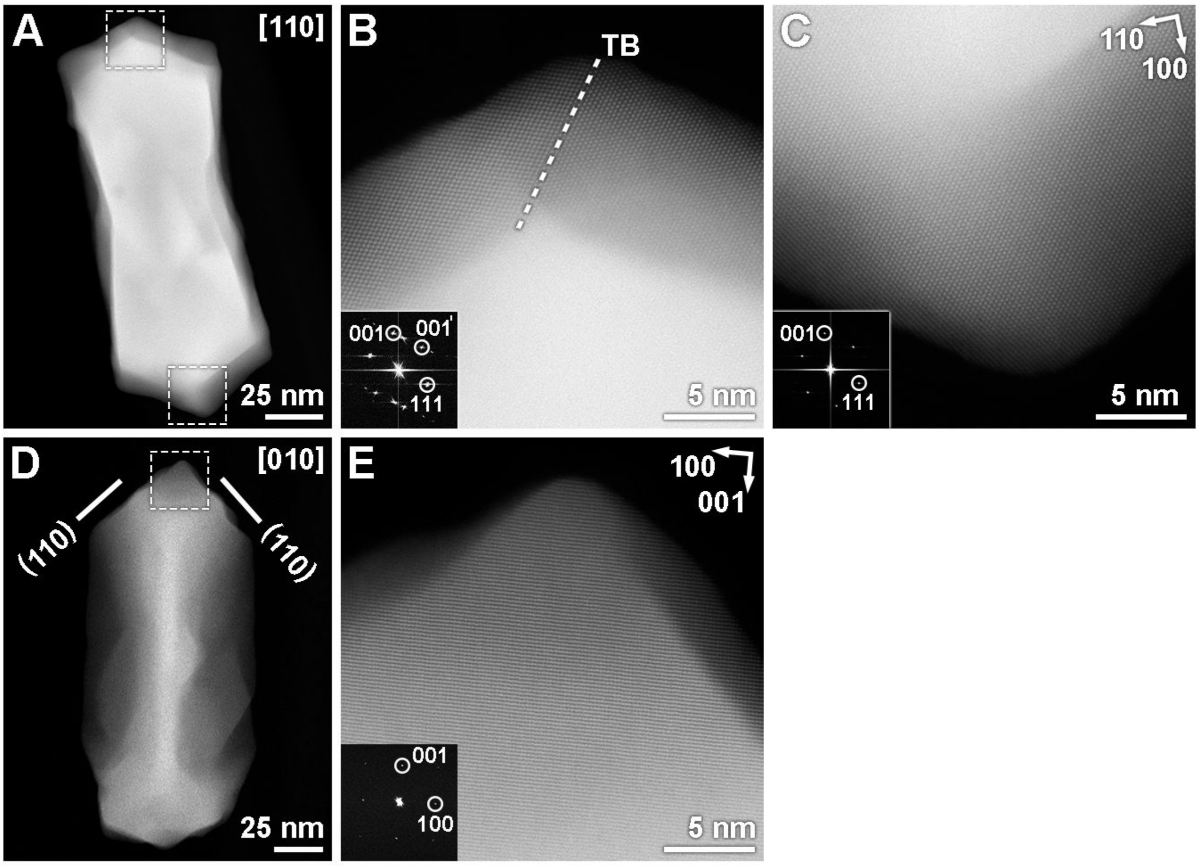

Figure S8. (A,D) HAADF-STEM images and (B,C,E) high resolution HAADF-STEM images of a chiral AuNR prepared with (*R*)-LipoCYS (similar to that in **Figure 3A**), taken along [110] and [100] zone axis. The insets in (B), (C) and (E) show fast Fourier transform (FFT) patterns along [110] and [100] directions for the fcc lattice of Au, which indicate that the tips are enclosed by {110} facets.

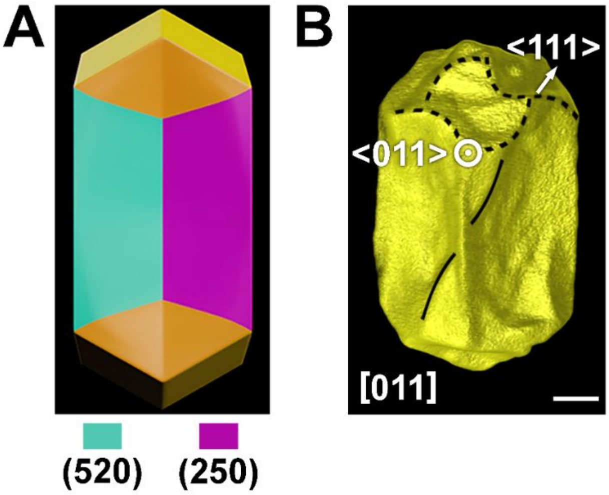

Figure S9. (A) Idealized surface morphology of a NP obtained using 20 μM (*R*)-LipoCYS, indicating that concave lateral facets of a NP consist of facets of the {520} family. (B) Electron tomography reconstruction of a NP obtained using 20 μM LipoCYS, tilted such that the viewing direction is parallel to the [011] direction, indicating twisted features forming around <011> and <111> corners (dashed black lines), similar to the particles reported by Lee *et al.*[10] Scale bar: 25 nm.

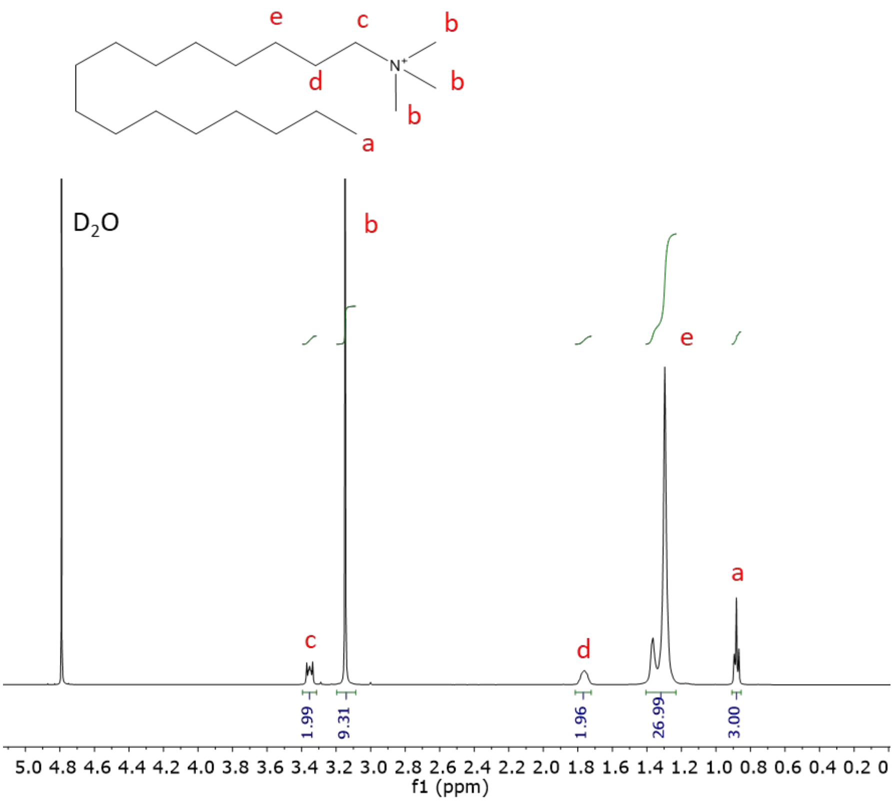

Figure S10. ^{1}H NMR spectrum (500 MHz, 298 K, D_2O) of a solution containing 44 mM CTAC and 90 µM (*R*)-LipoCYS.

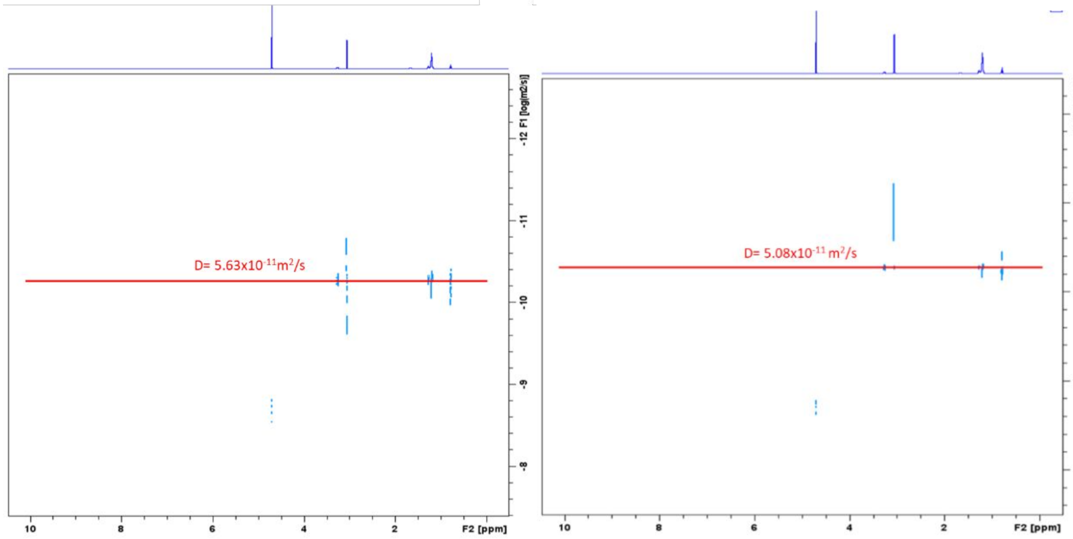

Figure S11. ^{1}H DOSY experiment (500 MHz, 298 K, D_2O) of a solution formed by (left panel) 44 mM CTAC, and (right panel) 44 mM CTAC and 90 µM (*R*)-LipoCYS. The measured diffusion coefficient for CTAC was 5.63×10^{-11} m^2/s, corresponding to a hydrodynamic diameter of 1.06 nm using the Stokes-Einstein equation. In the case of CTAC+LipoCYS, the diffusion coefficient is 5.08×10^{-11} m^2/s, corresponding to a hydrodynamic diameter of 1.8±0.6 nm.

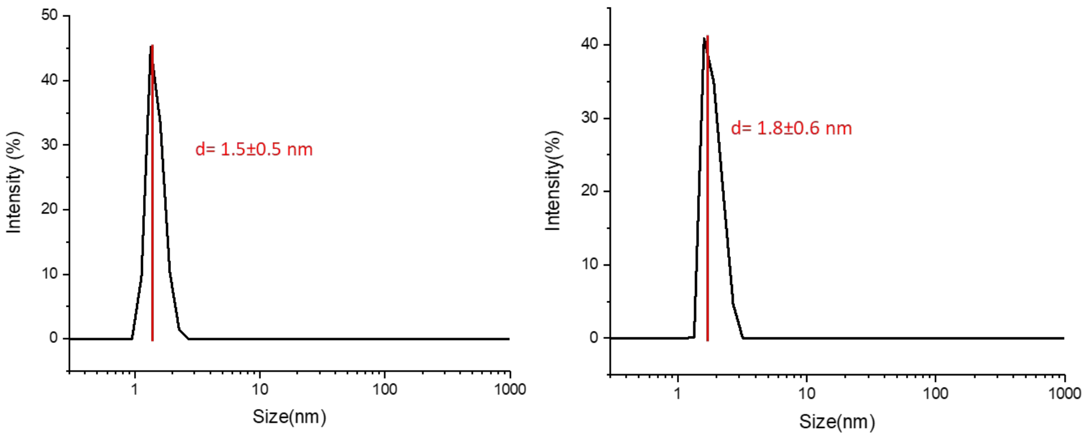

Figure S12. Particle size distribution measured by dynamic light scattering, of solutions containing (left panel) 44 mM CTAC, and (right panel) 44 mM CTAC and 90 µM (*R*)-LipoCYS.

Movie S1. Combined HAADF-STEM and electron diffraction (ED) tomography reconstruction of a selected NP, obtained using 20 µM (R)-LipoCYS.

References

1. Vora, H. D.; Johnson, M.; Brea, R. J.; Rudd, A. K.; Devaraj, N. K. Inhibition of NRAS Signaling in Melanoma through Direct Depalmitoylation Using Amphiphilic Nucleophiles. *ACS Chem. Biol.* **2020**, *15*, 2079-2086.
2. Gonzalez-Rubio, G. et al., Disconnecting Symmetry Breaking from Seeded Growth for the Reproducible Synthesis of High Quality Gold Nanorods. *ACS Nano* **2019**, *13*, 4424-4435.
3. Van Aarle, W.; Palenstijn, W. J.; De Beenhouwer, J.; Altantzis, T.; Bals, S.; Batenburg, K. J.; Sijbers, J. The ASTRA Toolbox: A Platform for Advanced Algorithm Development in Electron Tomography. *Ultramicroscopy* **2015**, *157*, 35–47.
4. Heyvaert, W.; Pedrazo-Tardajos, A.; Kadu, A.; Claes, N.; González-Rubio, G.; Liz-Marzán, L. M.; Albrecht, W.; Bals, S. Quantification of the Helical Morphology of Chiral Gold Nanorods. *ACS Materials Lett.* **2022**, *4*, 642-649.
5. Solís, D. M.; Taboada, J. M.; Obelleiro, F.; Liz-Marzán, L. M.; García de Abajo, F. J. Toward Ultimate Nanoplasmonics Modeling. *ACS Nano* **2014**, *8*, 7559-7570.
6. Solis, D. M.; Taboada, J. M.; Obelleiro, F. Surface Integral Equation Method of Moments with Multiregion Basis Functions Applied to Plasmonics. *IEEE Trans. Antennas Propag.* **2015**, *63*, 2141-2152.

7. Martín, V. F.; Solís, D. M.; Jericó, D.; Landesa, L.; Obelleiro, F.; Taboada, J. M. Discontinuous Galerkin Integral Equation Method for Light Scattering from Complex Nanoparticle Assemblies. *Opt. Express* **2023**, *31*, 1034-1048.
8. Johnson, P. B.; Christy, R. W. Optical Constants of the Noble Metals. *Phys. Rev. B* **1972**, *6*, 4370-4379.
9. Palik, E. D. Handbook of Optical Constants of Solids, Academic Press, San Diego 1998.
10. Lee, H.-E.; Kim, R. M.; Ahn, H.-Y.; Lee, Y. Y.; Byun, G. H.; Im, S. W.; Mun, J.; Rho, J.; Nam, K. T., Cysteine-Encoded Chirality Evolution in Plasmonic Rhombic Dodecahedral Gold Nanoparticles. *Nat. Commun.* **2020**, *11*, 263.
